# Beyond FimH: Diversity and Relevance of Carbohydrate‐Binding Fimbrial Proteins in *Escherichia coli*

**DOI:** 10.1002/cbic.202500433

**Published:** 2025-07-30

**Authors:** Oliwier R. Dulawa, Shane M. Coyle, Fiona Walsh, Trinidad Velasco‐Torrijos

**Affiliations:** ^1^ Department of Chemistry Maynooth University Maynooth W23 F2H6 Co. Kildare Ireland; ^2^ The Kathleen Lonsdale Institute for Human Health Research Maynooth University Maynooth W23 YCW7 Co. Kildare Ireland; ^3^ Department of Biology Maynooth University Maynooth W23 XY3X Co. Kildare Ireland

**Keywords:** adhesions, carbohydrates, *Escherichia coli*, lectins, membrane proteins

## Abstract

*Escherichia coli* (*E. coli*) is responsible for multiple diseases in humans and animals. Many of them are treated with antibiotics; however, the need for new therapies has led to research in alternative treatments. One such approach involves preventing the adherence of *E. coli* to host cells by inhibiting their adhesins. Adherence is a crucial step of pathogenesis, and bacterial lectins that recognize host glycans play major roles in host cell adhesion. In fact, lectins are the most common bacterial adhesins. The various pathogenic and nonpathogenic *E. coli* strains express a multitude of lectins, many of which are found on *E. coli* fimbriae. Current research on lectin inhibition using glycomimetics has produced many mannose‐based inhibitors of the uropathogenic *E. coli* fimbrial lectin FimH. However, only a limited number of synthetic inhibitors are reported for other lectins. In this review, many other cell surface adhesins of *E. coli* are discussed, focusing on fimbrial lectins. The types of *E. coli* strains they are found in, their carbohydrate targets, and their binding sites are also discussed. This review aims to highlight the many lectins that can become therapeutic targets to treat *E. coli* infections in addition to FimH.

## Graphical abstract

This review discusses fimbrial lectins found in *Escherichia coli* (*E. coli*) other than FimH, which could be targeted to treat *E. coli* pathogenicity as an alternative to antibiotic treatment.

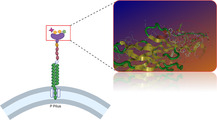

## 1 Introduction

### 1.1 The Relevance of Pili and Fimbriae

**Figure 1:**
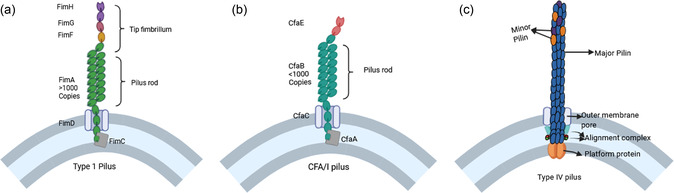
Schematic representation of: a) the structure of the type 1 pilus, an example of a CU pilus; b) the structure of the CFA/I pilus, an example of an alternate chaperone user pilus; and c) the structure of a type IV pilus in Gram‐negative bacteria. (a,b) Redrawn from^[^ ^9^ ^]^ and (c) Redrawn from.^[^ ^48^ ^]^ Created in BioRender. Coyle, S. (2025) https://BioRender.com/f33e154.

Pili and fimbriae are nonflagellar protein filaments that coat many bacterial cells (mostly Gram‐negative bacteria, but can also be found on Gram‐positive bacteria).^[^ ^1^, ^2^ ^]^ Although the terms are often used interchangeably, fimbriae are shorter, more numerous than pili and mediate bacterial adhesion. The longer conjugative pilus is the organelle responsible for transferring plasmids between donor and recipient cells.^[^ ^2^ ^]^ Pili and fimbriae serve several functions that include biofilm formation,^[^ ^3^ ^]^ conjugation,^[^ ^4^ ^]^ adhesion to environmental surfaces and host cells,^[^ ^5^ ^]^ motility,^[^ ^6^ ^]^ and transformation.^[^ ^7^ ^]^ They can also act as targets for bacteriophages.^[^ ^8^ ^]^ Fimbriae are polymeric structures consisting of the pilus rod, formed by repeating subunits, which are attached to the outer membrane.^[^ ^9^ ^]^ At the tip of the pilus rod there are adhesin proteins, which typically bind to specific receptors on the host. (**Figure** **1**) Carbohydrate epitopes in glycoproteins or glycolipids in the host cell surface are amongst the most important ones recognized by fimbrial adhesins.^[^ ^10^ ^]^

*Escherichia coli* (*E. coli*) strains are commensal Gram‐negative bacteria that are an important part of the normal gut microflora. However, there are also many pathogenic groups (or pathotypes) of *E. coli* that cause disease in humans and animals. Typically, three types of illnesses can result from infections by these pathotypes: 1) enteric or diarrhoeal disease (caused by enteropathogenic *E. coli* (EPEC), enterohaemorrhagic *E. coli* (EHEC), enterotoxigenic *E. coli* (ETEC), enteroaggregative *E. coli* (EAEC), enteroinvasive *E. coli* (EIEC), and diffusely adherent *E. coli* (DAEC)), 2) urinary tract infections (UTIs, caused by uropathogenic *E. coli* (UPEC)), and 3) sepsis or meningitis (caused by meningitis‐associated *E. coli* (MNEC)).^[^ ^10^, ^11^ ^]^ *E. coli* serotypes have traditionally been classified according to surface antigens: the O‐polysaccharide antigens, flagellar H‐antigens, and capsular K‐antigens, with over 180 different *E. coli* O‐groups and over 50 H‐types, which makes serotyping highly complex.^[^ ^12^ ^]^ There are ≈100 different fimbrial types expressed by *E. coli*.^[^ ^13^ ^]^ Serotypes are used for strain identification and tracking, while adhesion specificities are more directly related to pathogenic mechanisms and host tropism. Most pathogenic *E. coli* strains produce specific fimbrial adhesins, which represent essential colonization factors.^[^ ^14^ ^]^

One of the most thoroughly studied fimbrial adhesins is the adhesin of Type 1 pili (FimH), found predominantly in UPEC, but it is also present in other *E. coli* intestinal pathotypes such as EPEC and ETEC. FimH is also expressed in other pathogenic bacteria, including *Enterobacter cloacae, Citrobacter freundii*, *and Klebsiella pneumoniae*; it may also be expressed by some *Salmonella* species.^[^ ^15^, ^16^ ^]^ FimH binds to terminal α‐linked mannoses of the glycosylated receptor uroplakin on urinary epithelial cells or glycoproteins in the epithelial linings of the intestinal tract.^[^ ^17^, ^18^ ^]^ Due to its important role in the initial steps of infection, FimH has been thoroughly studied for the development of antiadhesion therapies,^[^ ^19^ ^]^ biofilm inhibition,^[^ ^20^ ^]^ and treatments for patients with Crohn's disease.^[^ ^21^, ^22^ ^]^ Much of this work has been done with synthetic derivatives of native carbohydrate drugs, termed glycomimetics.^[^ ^23^ ^]^ Numerous mannose (Man)‐based FimH antagonists have been reported and discussed previously in several recent reviews.^[^ ^24^, ^25^, ^26^ ^]^ Amongst those, the biphenyl mannosides by Ernst,^[^ ^27^ ^]^ Janetka, and Hultgren^[^ ^20^, ^28^ ^]^ have shown high potency and oral bioavailability in the treatment of UTIs. Remarkably, some of these compounds have progressed to different stages in clinical trials; these include the study lead by Takeda to prevent recurrence of Crohn's disease, which did not progress past Phase 2a,^[^ ^29^ ^]^ and the study involving FimH inhibitors optimized by Fimbrion and GSK, which has recently completed Phase 1b.^[^ ^30^ ^]^ In addition, Phase 2 clinical trials are being planned to evaluate the efficacy of a FimCH vaccine to prevent UTIs caused by UPEC.^[^ ^31^ ^]^

The intense research activity focusing on FimH inhibitors, along with the promising results obtained in this field so far, highlights the potential of fimbrial adhesins in the development of innovative antivirulence strategies. Importantly, there are many fimbrial proteins that have not yet been extensively investigated. While the structure and functionality of the different types of pili, fimbriae, and protein appendages in *E. coli* and other Gram‐negative bacteria have been discussed previously in some excellent reviews (see for example),^[^ ^15^, ^32^, ^33^ ^]^ here we will concentrate on the subset of these proteins that bind carbohydrates (other than FimH), as well as on the structures of their corresponding carbohydrate epitopes, when known. We will discuss their binding specificities, function, and potential as therapeutic targets for new strategies to treat *E. coli* infections.

### 1.2 *E. coli* Pili and Fimbriae Classifications

Pili and fimbria classification is quite complex and has changed significantly over the years.^[^ ^15^, ^34^ ^]^ *E. coli* pili and fimbriae are often classified into six groups, based on their biosynthetic pathways: 1) the chaperone‐usher (CU) pathway, 2) the alternate CU pathway (also called α fimbriae or class 5 pili), 3) type IV pili assembly, 4) curli synthesis, 5) type III secretion system pili, and 6) type IV secretion system pili (**Table** **1**).^[^ ^35^ ^]^ There is an alternate naming convention for some of these groups: the CU family is called classes 1,2, and 3; pili belonging to the alternate usher family have the name class 5 pili, type IV pili can also be called class 4 pili and curli can be referred to as class 6.^[^ ^36^ ^]^ CU pili are the most abundant group of surface filaments. In *E. coli*, this class would include Type I, Type III, Type IX, P, S, Dr and AUF pili subgroups.^[^ ^37^, ^38^ ^]^ Some of them, such as the type I and P pili, are tipped with carbohydrate‐binding proteins (lectins) like FimH and PapG, respectively, which specifically bind to glycans on host cells.^[^ ^39^ ^]^ Bundlin pilin proteins found in EPEC recognize and bind *N*‐acetyllactosamine (LacNAc) moieties at the surface of intestinal host cells.^[^ ^40^ ^]^ The class 5 pili include coli surface (CS) or colonization factor antigen (CFA) in ETEC. Class 5 pili assemble through a similar mechanism to the classical CU family and have similar structural characteristics; however, there is little sequence similarity between components of the two classes.^[^ ^41^ ^]^ Some CFA fimbriae adhere to sialylated glycoprotein on the small intestinal cell surface.^[^ ^42^ ^]^ *E. coli* also expresses aggregative, amyloid‐type curli fibers which promote cellular adhesion, invasion, and biofilm formation.^[^ ^43^ ^]^ Type III secretion systems are important in the pathogenesis of *E. coli*, although specific functional roles are still being elucidated.^[^ ^44^, ^45^ ^]^ Type IV pili are long, flexible filamentous structures that not only mediate the adherence of pathogenic *E. coli* to their hosts and other bacteria, but also are involved in biofilm formation, motility, and conjugation.^[^ ^46^, ^47^, ^48^ ^]^

**Table 1:** Confirmed pili‐based lectins found on *E. coli* (excluding FimH).

Pilus	Lectin adhesin	Alternate names	Assembly pathway	Ligand	Reference
F9 pilus Yde pilus^[^ ^49^ ^]^	FmlH	YdeQ^[^ ^15^ ^]^	CU^[^ ^39^ ^]^	TF antigen^[^ ^50^ ^]^ [SIF1]a) Terminal Gal/GalNAc^[^ ^50^ ^]^ [SIF2,3] GalNAc^[^ ^50^ ^]^ [SIF4] Terminal Gal‐β‐1,3‐GlcNAc^[^ ^51^ ^]^ [SIF5] Several synthetic ligands^[^ ^50^, ^52^, ^53^ ^]^ [SIF6]	[49]
P pilusc)	PapGI	PapG_J96_^[^ ^68^ ^]^ G‐I (GI) adhesin^[^ ^55^ ^]^	CU^[^ ^39^ ^]^	Gal‐α‐1,4‐Gal^[^ ^39^ ^]^ [SIF7] Several synthetic ligands^[^ ^65^, ^67^ ^]^ [SIF8]	[61]
P pilus KS71A^[^ ^164^ ^]^ KS71B^[^ ^164^ ^]^ F7 (F7_1_ and F7_2_)^[^ ^164^, ^165^ ^]^ F11^[^ ^56^ ^]^	PapGII	PapG_AD110_^[^ ^59^ ^]^ PapG_IA2_^[^ ^59^ ^]^ P adhesin^[^ ^60^ ^]^ G‐II (GII) adhesin^[^ ^55^ ^]^ FsoG^[^ ^75^ ^]^ FstG^[^ ^75^ ^]^	CU^[^ ^39^ ^]^	Gal‐α‐1,4‐Gal^[^ ^39^ ^]^ [SIF7] Several synthetic ligands^[^ ^65^ ^]^ [SIF8]	[61]
P pilus	PapGIII	PrsG_J96_^[^ ^59^ ^]^ PrsG^[^ ^58^ ^]^ F adhesin^[^ ^60^ ^]^ G‐III (GIII) adhesin^[^ ^55^ ^]^	CU^[^ ^39^ ^]^	Gal‐α‐1,4‐Gal^[^ ^39^ ^]^ [SIF7] GaINAc‐α‐1,3‐GaINAc^[^ ^166^ ^]^ [SIF9]	[61]
P pilus	PapGIV		CU^[^ ^39^ ^]^	GalNAc‐α‐1,3‐GalNAc‐β‐1,3‐Gal‐α‐1,4‐Gal‐β‐1,4‐Glc‐Cer (Globoside‐5)^[^ ^61^ ^]^ [SIF10]	[61]
P pilus	PapGV		CU^[^ ^39^ ^]^	GalNAc‐α‐1,3‐GalNAc‐β‐1,3‐Gal‐α‐1,4‐Gal‐β‐1,4‐Glc‐Cer (Globoside 5)^[^ ^61^ ^]^ d) [SIF10]	[61]
Plf	PlfGI, PlfGII		CU^[^ ^78^ ^]^	Globoside‐3, Globoside‐4, Globoside‐5^[^ ^78^ ^]^ [SIF10,11,12]	[78]
F1C	FocH	Type 1C^[^ ^39^ ^]^ KS71C^[^ ^167^ ^]^	CU^[^ ^39^ ^]^	GalNAc^[^ ^81^ ^]^ [SIF4] Gal^[^ ^81^ ^]^ [SIF13] Gal‐β‐1‐Cer^[^ ^167^ ^]^ [SIF14] Globotriaosylceramide (Globoside‐3)^[^ ^168^ ^]^ [SIF11] Asialo‐GM1, *K*_d_ = 109.1 ± 45.6 nM^[^ ^80^ ^]^ e) [SIF15] GalNAc‐β‐1,4‐Gal‐β^[^ ^84^ ^]^ [SIF16] Several synthetic ligands^[^ ^82^ ^]^ [SIF17]	[80]
S‐fimbriae Sfa‐I Sfa‐II^[^ ^84^ ^]^	SfaS	Sfa fimbriae	CU^[^ ^39^ ^]^	Glycoproteins containing sialic acid^[^ ^84^ ^]^ Neu5Ac‐α‐2,3‐Gal residues^[^ ^39^ ^]^ [SIF18] Neu5Ac‐α‐2,3‐Lac‐containing receptors, MIC = 0.3 mM^[^ ^79^, ^83^ ^]^ [SIF19]	[79]
F165_1_	N/Df)		CU^[^ ^39^ ^]^	GalNAc‐α‐1,3‐GalNAc‐β^[^ ^77^ ^]^ [SIF20] Human erythrocytes with blood group A_1_P_1_^[^ ^77^ ^]^ [SIF21,22] Gal‐α‐1,4‐Gal‐β^[^ ^77^ ^]^ [SIF23]	[76]
F6 (987 P)^[^ ^89^ ^]^ P987^[^ ^169^ ^]^	FasG		CU^[^ ^39^ ^]^	Glycolipids (Galactosylceramides and Sulfatide)^[^ ^89^, ^90^ ^]^ [SIF24]	[89]
F17 (Fy, FY, Att25)^[^ ^97^, ^170^ ^]^ More pili names based on major subunit as seen in the text	F17G‐F17aG^[^ ^95^ ^]^		CU^[^ ^15^ ^]^	GlcNAc‐β‐1,3‐Gal, *K*_d_ ≈ 0.66 × 10^−4^^[^ ^95^ ^]^ [SIF25] β‐GlcNAc, *K*_d_ ≈ 1.2 × 10^−3^^[^ ^94^, ^95^ ^]^ [SIF26] GlcNAc‐β‐1,2‐Man^[^ ^163^ ^]^ [SIF27] GlcNAc‐β‐1,4‐GlcNAc, *K*_d_ ≈ 10^−3^^[^ ^163^ ^]^ [SIF28] GlcNAc‐β‐1‐OMe^[^ ^163^ ^]^ [SIF29] GlcNAc‐β‐1‐SeMe^[^ ^163^ ^]^ [SIF30]	[95]
F17 (Fy, Att25, Vir adhesin F17‐like)^[^ ^97^, ^170^ ^]^	F17G‐F17bG^[^ ^95^ ^]^		CU^[^ ^15^ ^]^	GlcNAc‐β‐1,3‐Gal, *K*_d_ ≈ 10^−3^^[^ ^95^ ^]^ [SIF25] β‐GlcNAc, *K*_d_ ≈ 10^−3^^[^ ^94^, ^95^ ^]^ [SIF26] GlcNAc‐β‐1,2‐Man^[^ ^163^ ^]^ [SIF27] GlcNAc‐β‐1,4‐GlcNAc^[^ ^163^ ^]^ [SIF28]	[95]
F17 (Fy, Att25, 20 K, G)^[^ ^97^, ^170^ ^]^	F17G‐F17cG^[^ ^95^ ^]^	GafD^[^ ^94^ ^]^	CU^[^ ^15^ ^]^	GlcNAc‐β‐1,3‐Gal^[^ ^95^ ^]^ [SIF25] β‐GlcNAc^[^ ^94^, ^95^ ^]^ [SIF26]	[95]
F17 (Fy, Att25, Att111, F111)^[^ ^97^, ^170^ ^]^	F17G‐F17dG^[^ ^95^ ^]^		CU^[^ ^15^ ^]^	GlcNAc‐β‐1,3‐Gal^[^ ^95^ ^]^ [SIF25] β‐GlcNAc^[^ ^94^, ^95^ ^]^ [SIF26]	[95]
F17 (Fy, Att25)^[^ ^97^ ^]^	F17G‐F17eG^[^ ^95^ ^]^		CU^[^ ^15^ ^]^	GlcNAc‐β‐1,3‐Gal, *K*_d_ ≈ 0.28 × 10^−4^^[^ ^95^ ^]^ [SIF25] β‐GlcNAc^[^ ^94^, ^95^ ^]^ [SIF26]	[95]
F17 (Fy, Att25)^[^ ^97^ ^]^	F17G‐F17fG^[^ ^163^ ^]^		CU^[^ ^15^ ^]^	GlcNAc‐β‐1,3‐Gal, *K*_d_ ≈ 10^−4^^[^ ^95^ ^]^ [SIF25] β‐GlcNAc, *K*_d_ ≈ 10^−3^^[^ ^94^, ^95^ ^]^ [SIF26]	[163]
Ucl Fimbriae	UclD	F17‐like fimbriae^[^ ^99^ ^]^	CU^[^ ^99^ ^]^	Sialyllacto‐*N*‐fucopentose VI^[^ ^99^ ^]^ [SIF31]	[99]
F4‐F4_ab_^[^ ^103^ ^]^ K88ab^[^ ^101^ ^]^	FaeG		CU^[^ ^39^ ^]^	Lac^[^ ^103^ ^]^ [SIF32] Gal‐α‐1,3‐Gal^[^ ^108^ ^]^ [SIF33] An intestinal mucin‐type Neu5Acglycoprotein^[^ ^105^ ^]^ Galactosamine (GalN)^[^ ^126^ ^]^ [SIF34] Transferrin^[^ ^104^ ^]^ Gal‐α‐1,4‐Gal‐β‐1‐Cer^[^ ^103^ ^]^ [SIF35] Gal‐α‐1,4‐Gal‐β‐1,4‐Glc‐β‐1‐Cer^[^ ^103^ ^]^ [SIF36]	[103]
F4‐F4_ac_^[^ ^103^ ^]^ K88ac^[^ ^101^ ^]^	FaeG		CU^[^ ^39^ ^]^	Gal‐β‐1,3‐GalNAc^[^ ^109^ ^]^ [SIF37] Fuc‐α‐1,2‐Gal‐β‐1,3/4‐GlcNAc^[^ ^109^ ^]^ [SIF38,39] An intestinal mucin‐type Neu5Acglycoprotein^[^ ^105^ ^]^ Gal‐β‐1,3‐GlcNAc^[^ ^80^ ^]^ [SIF40] Gal‐α‐1,3‐Gal^[^ ^80^ ^]^ [SIF33] Asialo‐GM1^[^ ^80^ ^]^ [SIF15]	[103]
F4‐F4_ad_^[^ ^103^ ^]^ K88ad^[^ ^101^ ^]^	FaeG		CU^[^ ^39^ ^]^	IGLad glycosphingolipid (neolactotetraosylceramide)^[^ ^101^, ^106^ ^]^ [SIF41] Lac^[^ ^103^ ^]^ [SIF32] (This protein contains a galactose binding site^[^ ^103^ ^]^)	[103]
F‐18 fimbriae F18ac F18ab	FedF	8813, 2134 P, F107, Av24^[^ ^97^, ^110^ ^]^	CU^[^ ^39^ ^]^	Glycosphingolipids containing Fuc‐α‐1,2‐Gal‐β‐1,3‐GlcNAc, Gal‐α‐1,3‐(Fuc‐α‐1,2)‐Gal‐β‐1,3‐GlcNAc or GalNAc‐α‐1,3‐(Fuc‐α‐1,2)‐Gal‐β‐1,3‐GlcNAc^[^ ^112^ ^]^ [SIF38,42,43] Sulfated LacNAc^[^ ^95^ ^]^ Gal‐β‐1,4‐GlcNAc sulfated at positions 3', 6' and 6^[^ ^95^ ^]^ [SIF44]	[112]
F5 fimbriae	FanC	K99^[^ ^116^ ^]^	CU^[^ ^39^ ^]^	Neu5Ac‐α‐2,3‐Gal‐β‐1,3‐GlcNAc^[^ ^80^ ^]^ [SIF45] Other sialoglycolipids^[^ ^171^ ^]^ Neu5Gc‐GM3^[^ ^119^ ^]^ [SIF46] Neu5Gc‐paragloboside^[^ ^119^ ^]^ [SIF47]	[80]
ECP fimbriae	EcpD	Meningitis‐associated and temperature‐regulated fimbriae (Mat)^[^ ^80^ ^]^ Yag^[^ ^15^ ^]^	CU^[^ ^15^ ^]^	Gal‐α‐1,6‐Glc^[^ ^80^ ^]^ [SIF48] Asialo‐GM1^[^ ^80^ ^]^ Blood group B trisaccharide^[^ ^80^ ^]^ [SIF49] GlcNAc‐β‐1,6‐Gal‐β‐1,4‐GlcNAc^[^ ^80^ ^]^ [SIF50] l‐Arabinosyl residues,^[^ ^121^ ^]^ [SIF51]	[120]
Yad	YadC		CU^[^ ^39^ ^]^	Xylose^[^ ^124^ ^]^ [SIF52]	[124]
Yqi fimbriae	ExPEC adhesin I	Yqi adhesin	CU^[^ ^39^ ^]^	Gal‐β‐1,4‐GlcNAc‐β‐1,3‐(GlcNAc‐β‐1,6)‐Gal‐β‐1,4‐GlcNAc‐Sp2 spacer,^[^ ^80^ ^]^ g) [SIF53] GlcNAc‐β‐1,3‐Gal‐β‐1,4‐GlcNAc‐β‐Sp3 spacer,^[^ ^80^ ^]^ [SIF54] Gal‐β‐1,3‐(Fuc‐α‐1,4)‐GlcNAc‐β‐Sp3,^[^ ^80^ ^]^ g) [SIF55] Fuc‐α‐1,2‐(Gal‐α‐1,3)‐Gal‐β1,4‐GlcNAc‐β‐Sp3,^[^ ^80^ ^]^ g) [SIF56] Fuc‐α‐1,3‐(Gal‐α‐1,3‐Gal‐β1,4)‐GlcNAc‐β‐Sp3,^[^ ^80^ ^]^ g) [SIF57] Has a preference for terminal GlcNAc.^[^ ^80^ ^]^ [SIF58]	[80]
AF/R1	AfrD/AfrE^[^ ^172^ ^]^		CU^[^ ^39^ ^]^	Galactosylceramide (Galβ‐1‐1‐Cer)^[^ ^125^ ^]^ [SIF59]	[125]
F42 fimbriae	F42 lectin		CU^[^ ^173^ ^]^	GalNAc^[^ ^126^ ^]^ [SIF4]	[126]
CFA/I	CfaB/CfaE	F2 (antigen), (encoded by *cfa*)^[^ ^39^ ^]^	Class 5^[^ ^39^ ^]^	Isoglobotriaosylceramide^[^ ^132^ ^]^ [SIF60] Gal‐α‐1,3‐Gal‐α‐1,3‐Gal‐β‐1,4‐Glc‐β‐1‐Cer^[^ ^132^ ^]^ [SIF61] Lactosylceramide^[^ ^132^ ^]^ [SIF62]	[132]
CS2	CotD^[^ ^135^ ^]^	CFA/II^[^ ^135^ ^]^ F3^[^ ^173^ ^]^	Class 5^[^ ^39^ ^]^	Neu5Gc, IC_50_ = 17.3 mM^[^ ^134^ ^]^ [SIF63] Neu5Ac, IC_50_ = 32.9 mM^[^ ^134^ ^]^ [SIF64] Neu5Ac‐Lac (linkage not specified), IC_50_ = 6.2 mM^[^ ^134^ ^]^ [SIF65]	[135]
CS4 *Csa* ^[^ ^132^ ^]^	CsaE	CFA/IV^[^ ^135^ ^]^	Class 5^[^ ^39^ ^]^	Isoglobotriaosylceramide^[^ ^132^ ^]^ [SIF60] Gal‐α‐1,3‐Gal‐α‐1,3‐Gal‐β‐1,4‐Glc‐β‐1‐Cer^[^ ^132^ ^]^ [SIF61] Lactosylceramide^[^ ^132^ ^]^ [SIF62]	[132]
CS7		334 A fimbriae^[^ ^174^ ^]^	Class 5 due to similarity with CS5^[^ ^140^ ^]^	Isoglobotriaosylceramide^[^ ^132^ ^]^ [SIF60] Lactosylceramide^[^ ^132^ ^]^ [SIF62] Neolactotetraosylceramide^[^ ^132^ ^]^ [SIF41]	[132]
CS3		CFA/II^[^ ^135^ ^]^ F3^[^ ^173^ ^]^	CU^[^ ^39^ ^]^	GM1^[^ ^139^ ^]^ [SIF66] Asialo‐GM1^[^ ^139^ ^]^ [SIF15] GM2^[^ ^139^ ^]^ [SIF67]	[139]
CS21 (longus)	LngA		Type IV^[^ ^142^ ^]^	Sialic acids (including Neu5Ac)^[^ ^142^ ^]^	[142]
N/D	N/D	Fimbrial		Gal [SIF13]	[157]
Type 3 fimbriae	MrkD		CU^[^ ^39^ ^]^	Type V collagen^[^ ^127^ ^]^ (highly glycosylated) Mannan (variant‐dependent)^[^ ^129^ ^]^ Has a lectin domain^[^ ^129^ ^]^	[129]

a) The chemical structures, as well as SNFG (symbol nomenclature for Glycans) representation of selected ligands are provided in the Supporting Information Figures (SIF).

b) The abbreviations for monosaccharides are as follows: d‐Galactose = Gal, *N*‐Acetyl‐d‐galactosamine = GalNAc, *N*‐Acetyl‐d‐glucosamine = GlcNAc, d‐Glucose = Glc, *N*‐Acetyl‐d‐neuraminic acid = Neu5Ac, d‐Lactose = Lac, d‐Mannose = Man, d‐Galactosamine = GalN, l‐Fucose = Fuc and *N*‐Glycolyl‐d‐neuraminic acid = Neu5Gc. In addition, Cer = ceramide. d or l configuration is only explicitly noted for l‐fucose and l‐arabinose.

c) P pili were given antigen numbers F7 to F16.^[^ ^173^ ^]^

d) For the purpose of this table, Globoside‐5 is treated as the Forsmann antigen, as indicated in the references provided in the Table. Globoside‐5 has also been referred to as Gal‐β‐1,3‐GalNAc‐β‐1,3‐Gal‐α‐1,4‐Gal‐β‐1,4‐Glc‐Cer, distinct from the Forsmann antigen.^[^ ^70^ ^]^

e) IC_50_, *K*_d_, or minimum inhibitory concentration (MIC) values of ligands are given when known.

f) Not described.

g) Sp2 and Sp3 refer to the chemical structure of the spacer groups linked to the glycans: Sp2 is ‐(CH_2_)_3_‐NH‐ and Sp3 is ‐(CH_2_)_5_‐NH‐.^[^ ^175^ ^]^

## 2 Chaperone‐Usher (CU) Fimbrial Lectins in *E. coli*

Fimbrial adhesins assembled through the CU pathway are the most abundant class of carbohydrate‐binding (lectin) adhesins in *E. coli*. Type I pili and their mannose‐recognizing adhesin FimH, the best studied lectin adhesin in *E. coli*, also belongs to this category.^[^ ^17^, ^24^ ^]^ Many other CU adhesins have been confirmed to have lectin activity, while others have been proposed as lectins according to their sequence analysis. Aside from FimH, this discussion will cover several other significant CU adhesins, some of which may serve as potential therapeutic targets.

### 2.1 FmlH

**Figure 2:**
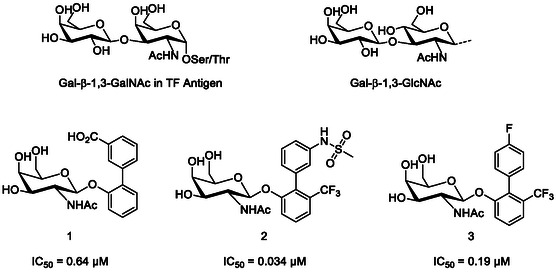
Top: Chemical structure of the carbohydrate epitopes recognized by FmlH, e.g., Thomsen–Friedenreich (TF) antigen and terminal Gal‐β‐1,3‐GlcNAc and Bottom: Chemical structures and binding affinities of glycomimetic ligands for FmlH developed by Hultgren and Janetka.^[^ ^50^, ^52^, ^53^ ^]^

FmlH is the adhesin found in F9 pili. The F9 pilus, also referred to as the Fml/Yde pilus, is significant for the colonization of the kidney and bladder in chronic UTIs. It is the pilus most closely related to the Type 1 pilus, which is also implicated in UTIs.^[^ ^49^ ^]^ FmlH, (also called YdeQ), is the tip adhesin that facilitates binding to host tissues.^[^ ^15^ ^]^ In the kidney, FmlH exhibits a high affinity for terminal Gal‐β‐1,3‐GalNAc epitopes, known as the Thomsen–Friedenreich (TF) antigen. Additionally, FmlH binds to terminal galactose (Gal) and *N*‐acetylgalactosamine (GalNAc) residues, particularly in the kidney and inflamed bladder.^[^ ^50^ ^]^ FmlH can also recognize Gal‐β‐1,3‐GlcNAc glycans and lacto‐*N*‐tetraose (**Figure** **2**). F9 genes are highly prevalent in UPEC isolates from urosepsis patients. Furthermore, 90% of the strains in a well‐defined *E. coli* reference (ECOR) collection express at least one F9 gene.^[^ ^51^ ^]^

FmlH has emerged as a potential target to attenuate virulence factors in UTIs, which has prompted the development of several synthetic ligands, some of which are shown in Figure 2. Hultgren and Janetka initially reported compound **1**, the most potent of a small set of biphenyl Gal and GalNAc glycomimetics.^[^ ^52^ ^]^ The affinity of compound **1** for FmlH (IC_50_ = 0.64 μM) was subsequently improved using X‐ray structure‐guided design, yielding compounds such as **2** (IC_50_ = 0.034 μM), which has been cocrystallized with FmlH (**Table** **2**). Compound **2** also exhibited excellent metabolic stability in mouse plasma and liver microsomes.^[^ ^50^ ^]^ Further optimization led to the development of several orally bioavailable FmlH ligands like compound **3**, albeit with a slightly lower affinity for FmlH (IC_50_ = 0.19 μM, calculated by ELISA competition assays).^[^ ^53^ ^]^ The overlay of the structures of compounds **2** and **3** cocrystallized with FmlH shows high similarity with respect to the GalNAc sugar binding, however the positions of the biaryl rings differ significantly. Tyrosine (Tyr46) and arginine (Arg142) side chains contribute to the aglycones binding through edge‐to‐face π‐stacking, electrostatic and hydrophobic interactions. The stronger interaction between the sulfonamide in **2** and FmlH accounts for the potency difference between the two compounds. The availability of structural data (such as the FmlH crystal structures with compounds **2** and **3**) enables the use of several computational techniques, such as fragment‐based e‐pharmacophore virtual screening protocols, which allows for the design of potential FmlH ligands.^[^ ^54^ ^]^

**Table 2:** Fimbrial lectins on *E. coli* with synthetic ligands and/or experimental 3D structures. FimH is not included.

Pilus	Adhesin	Crystal or Cryo‐EM structure (with natural ligand)	Synthetic ligand	Crystal structure (with synthetic ligand)	Reference
F9 pilus^a^^,^ b)	FmlH	PDB ID: 6aox^[^ ^52^ ^]^ c)	Yes, highest affinity IC_50_ = 34 nM	PDB ID: 6maw^[^ ^50^ ^]^	[50, 52]
P pilus	PapGI	N/D	Yes, highest affinity IC_50_ = 2 μM	N/D	[67]
P pilus	PapGII	PDB ID: 1j8r^[^ ^63^ ^]^	Yes, highest affinity IC_50_ = 68 μM	PDB ID: 4z3g^[^ ^69^ ^]^	[65, 69]
F1C	FocH	N/D	Yes, highest affinity IC_50_ = 15 μM		[82]
F17	F17G‐F17aG^[^ ^95^ ^]^	PDB ID: 3f6j^[^ ^95^ ^]^	N/D	PDB ID:1zpl^[^ ^171^ ^]^	[95, 171]
F17	F17G‐F17bG^[^ ^95^ ^]^	PDB ID: 4k0o^[^ ^95^ ^]^	N/D	N/D	[95]
F17	F17G‐F17cG (GafD)^[^ ^95^ ^]^	PDB ID: 1oio^[^ ^100^ ^]^	N/D	N/D	[96]
F17	F17G‐F17eG^[^ ^95^ ^]^	PDB ID: 2bsb^[^ ^95^ ^]^	N/D	N/D	[95]
F17	F17G‐F17fG^[^ ^171^ ^]^	PDB ID: 1zk5^[^ ^171^ ^]^	N/D	N/D	[171]
Ucl Fimbriae	UclD	PDB ID: 7mzp,^[^ ^99^ ^]^ d)	N/D	N/D	[99]
F4	F4_ab_	PDB ID: 4we2^[^ ^103^ ^]^	N/D	N/D	[103]
F4	F4_ac_	PDB ID: 4wen,^[^ ^176^ ^]^ d)	N/D	N/D	[176]
F4	F4_ad_	PDB ID: 4wei^[^ ^106^ ^]^	N/D	N/D	[106]
F18	FedF	PDB ID: 4b4q^[^ ^113^ ^]^	N/D	N/D	[113]
CFA/I	CfaB/CfaE	PDB ID: 6nrv^[^ ^177^ ^]^ d)	N/D	N/D	[177]
CS6	CssB	PDB ID: 4b9g,^[^ ^178^ ^]^ d)	N/D	N/D	[178]
Type 3	MrkD	PDB ID: 3u4k,^[^ ^179^ ^]^ d) (crystallized from a different species.)	N/D	N/D	[129, 179]

a) In the case where these lectins have multiple experimental 3D structures, a representative structure was selected.

b) FimH has been excluded, but it has multiple crystal structures and synthetic ligands, which are beyond the scope of this review. For some excellent recent reviews see refs. [24, 25, 26].

c) Protein Data Bank Identifier (PDB ID).

d) These are unbound protein structures because 3D structures that contain ligands are unavailable for these proteins on the protein data bank (PDB).

### 2.2 PapG

PapG adhesins are arguably the most studied type of *E. coli* adhesins after FimH. PapG adhesins are found on the tip of P‐fimbriae (pyelonephritis‐associated pili) in UPEC.^[^ ^55^ ^]^ They bind galabiose (Gal‐α‐1,4‐Gal) containing glycolipids such as globosides.^[^ ^56^ ^]^ Five PapG classes encoded by five different alleles of the PapG gene have been reported, each with different glycan binding sites.^[^ ^57^ ^]^ Among the P‐type fimbrial adhesins in *E. coli*, PapGI‐III is the most thoroughly studied. The different classes of PapGI‐IV have been given alternative names (see Table 1). PapGI (or GI adhesin), also referred to as PapG_J96_, is an adhesin associated with P pili, which are classified under the F13 serotypes.^[^ ^58^ ^]^ PapGII (or GII adhesin) is also known as PapG_AD110_ (for the F7_2_ serotype) or PapG_IA2_ (for the F11 serotype), as they are encoded by these sequences; PapG_AD110_ and PapG_IA2_ have almost identical glycolipid binding profiles.^[^ ^59^ ^]^ PapGIII has been called PrsG_J96_ or PrsG, and is present on the F13 serotype.^[^ ^60^ ^]^ This variant of PapG has been referred to as the F adhesin.^[^ ^48^ ^]^

Fimbrial adhesins in UPEC strains are effective virulence factors that are critical for bacterial pathogenesis initiation in UTIs. In a recent study by Golpasand et al., the analysis of fimbrial adhesin gene (FAG) patterns in UPEC strains isolated from UTI patients showed the highest frequency corresponding to FimH (found in 93.3% of isolates), with PapG found in 37.5% of the isolated strains; of this 37.5%, the prevalence of PapGI, PapGII, and PapGIII genes was identified as 2.9%, 30.8%, and 3.8% (PapGII > PapGIII > PapGI), respectively.^[^ ^37^ ^]^ PapGII has also been shown to be a significant risk factor for progression from UTI to bacteremia^[^ ^61^ ^]^ and bloodstream invasions.^[^ ^62^ ^]^ PapGII preferentially binds globoside GbO4, a glycolipid isoreceptor of the human kidney.^[^ ^56^ ^]^

**Figure 3:**
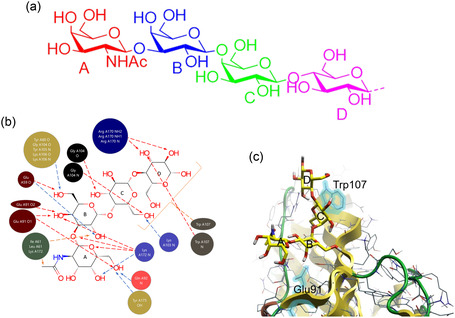
a) Chemical structure of the tetrasaccharide (GalNAc‐β‐1,3‐Gal‐α‐1,4‐Gal‐β‐1,4‐Glc) component of GbO4, the natural ligand for PapGII: reducing end Glc (residue D, pink), Gal (residue C, green), Gal (residue B, blue), and terminal GalNAc (residue A, pink); b) graphical representation of interactions between protein and receptor. Direct polar interactions are indicated by red arrows. Water mediated interactions are indicated by blue arrows. Brackets and arrows in orange indicate contacts with aromatic/hydrophobic platforms. Redrawn from^[^ ^63^ ^]^ using PDB ID: [1j8r] created with Flare from Cresset;^[^ ^159^, ^160^, ^161^, ^162^ ^]^ and c) a graphical representation of the binding site of the PapGII adhesin with the tetrasacharide component of GbO4 from X‐ray structure PDB ID: 1j8r. The Glu91 and Trp107 important for binding are shown. Redrawn from^[^ ^63^ ^]^ using PDB ID: [1j8r] created with Flare from Cresset.^[^ ^159^, ^160^, ^161^, ^162^ ^]^

The X‐ray crystal structure of PapGII bound to GbO4 (GalNAc‐β‐1,3‐Gal‐α‐1,4‐Gal‐β‐1,4‐Glc linked to ceramide (Cer)) (**Figure** **3**, showing the tetrasaccharide component of GbO4)^[^ ^63^ ^]^ has provided valuable information on the molecular interactions required for stable adhesin‐carbohydrate epitope binding. As shown in Figure 3, the tetrasaccharide binds to PapG in a V‐shape, with reducing end Glc (residue D) and the Gal residue next to it (C) forming one branch of the V, and the following Gal (B) and GalNAc (A) residues forming the other branch. The crystal structure reveals several water molecules in the binding site which bridge contacts between the carbohydrate ligand and the protein. H‐bonds, hydrophobic, and aromatic contacts between tryptophan Trp107 and Gal‐β‐1,4‐Glc are essential for the binding of PapGII to the glycan. Another key residue for binding is that of arginine Arg170, which makes interactions with the O2 and O3 hydroxyl groups of residue D. Glu 59 interacts with the hydroxyl in O6 in residue C and makes water‐mediated contact with hydroxyl in O2 in residue B. In residue B, the O4 hydroxyl participates in H‐bonding between the changed Glu91 and Lys172 side chains, and its O6 hydroxyl forms an H‐bond with the main chain nitrogen of Gly104. The binding of residue A is also mediated by Lys172, which interacts with the O5 and the methyl in the acetyl group in the GalNAc and makes water‐mediated contacts with the O4 hydroxyl. Glu91 makes direct H‐bond interactions with the O6 hydroxyl. In addition to the crystallographic structures, the solution structure of the adhesin domain in PapGII from UPEC and its recognition of Gal‐α‐1,4‐Gal (galabiose) have been investigated by nuclear magnetic resonance (NMR).^[^ ^64^ ^]^ This study showed that although the PapGII adhesin shares some structural similarities with FimH, the carbohydrate‐binding domain is located in one side of PapGII (in contrast to FimH, where carbohydrate recognition occurs mainly at the tip of the structure). This study also highlighted that Ile173, Lys172, Tyr166, Glu109, Leu102, and Ser89 residues are surface‐exposed and were most strongly affected during the NMR titration with galabiose. It has been proposed that PapG class specificity may be related to variations in the residue type in these positions.

These studies provided detailed knowledge of the intricate interactions between PapGII adhesins and their carbohydrate receptors, which are highly valuable for the design of synthetic ligands and glycomimetic compounds targeting these proteins. Although the optimal carbohydrate epitope for PapGII is the tetrasaccharide found in GbO4, most synthetic ligands for PapG class I and II are based on the galabiose disaccharide.

**Figure 4:**
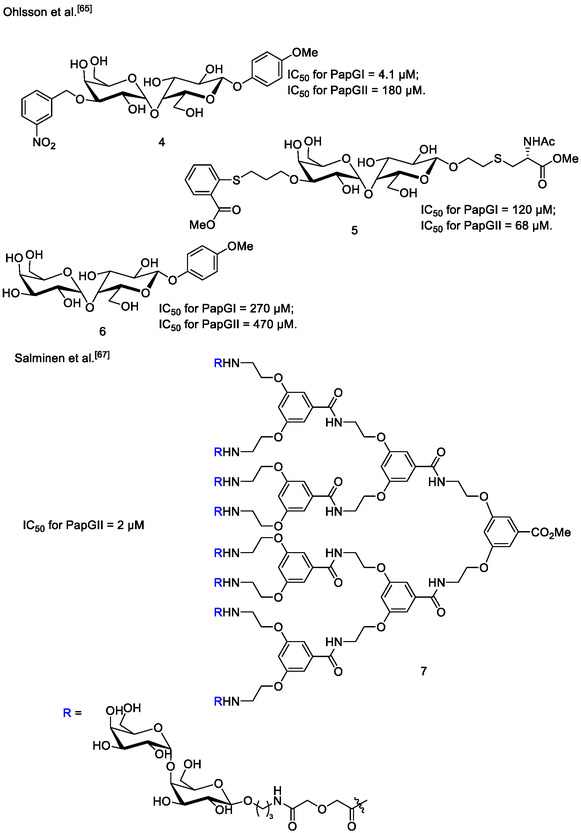
Chemical structures of selected synthetic ligands for PapG.^[^ ^65^, ^67^ ^]^

Ohlsson et al. investigated synthetic derivatives based on a galabiose core as inhibitors of PapG class I and II.^[^ ^54^ ^]^ The inhibitors were discovered by screening small libraries of galabiose functionalized at the O1 and O3′ positions. PapGI binding is favored by hydrophobic substituents at the O1 and is not hampered by modifications at the O3′ position of galabiose. On the other hand, the binding site of PapGII extends beyond the galabiose disaccharide, so the introduction of substituents that interact with the lectin in a favorable manner can offset the absence of additional carbohydrate moieties. Compound **4** (**Figure** **4**), which features aromatic substituents at both ends of the galabiose core, was found to inhibit PapGI with an IC_50_ of 4.1 μM by hemagglutination inhibition assay, making it 20–30 times more potent than the natural ligand GbO4. The relative potency of Compound **4** to GbO4 was found by comparing its hemagglutination inhibition assay results to the hemagglutination inhibition assay results of a reference with a known IC_50_ for inhibiting adhesion to GbO4.^[^ ^65^, ^66^ ^]^ Compound **5** (Figure 4), with an IC_50_ of 68 μM as determined by hemagglutination inhibition assay, is still the most potent reported synthetic inhibitor of PapGII and has an activity comparable to the tetrasaccharide in GbO4. Interestingly, compound **6,** functionalized only at the O1 position, retains significant affinity for both PapGI and PapGII adhesins (IC_50_ of 11 and 110 μM by hemagglutination inhibition assay, respectively). This can be accounted for by aromatic stacking interactions between the aglycon and conserved Trp107 residue, located near galabiose O1, as found in the crystal structure of the PapGII.

Some synthetic ligands and glycomimetics that target different PapG variants have been reported. Salminen et al. reported a series of mono and multivalent galabiose derivatives as inhibitors of adhesion of *E*. *coli* PapG_J96_ (class I) (Figure 4). Interestingly, while inhibition of adhesion was not strongly affected by multivalency, the octavalent compound **7** was found to be the most effective inhibitor, with the lowest IC_50_ (2 μM) for inhibition of PapGI reported to date (though a different assay was used to determine this IC_50_ as compared to the previously discussed inhibitors of PapGI). This value was determined using a live‐bacteria application of surface plasmon resonance (SPR), to mimic the flow conditions of natural infections. It showed a minimum inhibitory concentration (MIC) value of 9 μM as determined by hemagglutination assay.^[^ ^67^ ^]^

**Figure 5:**
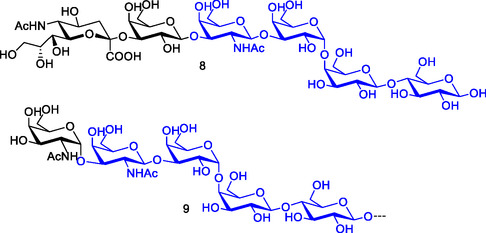
Chemical structures of the P blood group antigen hexasaccharide Neu5Ac‐α‐2,3‐Gal‐β‐1,3‐Gal*N*Ac‐β‐1,3‐Gal‐α‐1,4‐Gal‐β‐1,4‐Glc **8,** a natural ligand for PapGII^[^ ^69^ ^]^ and Forssman antigen **9** (featuring terminal GaINAc‐α‐1,3‐GaINAc).^[^ ^70^ ^]^ The structure of the GbO4 tetrasaccharide is shown in blue.

Although PapGII preferentially binds to the globoside GbO4, early studies by Stapleton et al. reported increased affinity for the natural sialosyl galactosyl globoside (SGG), which features the P blood group antigen hexsaccharide (Neu5Ac‐α‐2,3‐Gal‐β‐1,3‐Gal*N*Ac‐β‐1,3‐Gal‐α‐1,4‐Gal‐β‐1,4Glc **8,** **Figure** **5**)^[^ ^68^ ^]^ (Neu5Ac is *N*‐acetylneuraminic acid). Using isothermal titration calorimetry (ITC), Navarra et al. determined dissociation constants (*K*_D_) and thermodynamic parameters of the binding between PapGII and several carbohydrate ligands including hexasacharide **8** and the GbO4 tetrasaccharide (Figure 5).^[^ ^69^ ^]^ Hexasaccharide **8** had threefold higher affinity than the GbO4 tetrasaccharide (*K*_D_ = 21.9 and 59.1 μM, respectively). Notably, the structure of PapGII cocrystallized with **8** did not show a direct interaction of the additional Neu5Ac‐α‐2,3‐Gal‐β moiety with the protein. Alongside with the experimental ITC measurements, the authors of this study carried out molecular dynamic simulations and proposed that the presence of extended solvation shells at the surface of both protein and ligand accounts for the additional affinity. Upon ligand binding to the carbohydrate recognition domain, the disaccharide moiety in **8** is close to the PapGII surface, which forces both ligand and protein to release water molecules from their outer solvation shells. This entropic contribution due to the desolvation of nonbinding components of the saccharide ligands may be useful in ligand design to improve carbohydrate‐lectin interactions. Interestingly, this study suggests that the main contribution to the binding of the glucose unit at the reducing end in the GbO4 tetrasaccharide is due to lipophilic interactions established by the hydrophobic β‐face, and its replacement by aromatic rings (as in some synthetic ligands such as **6**) maintains this interaction, although with reduced desolvation cost.

PapGIII, also known as the F adhesin,^[^ ^60^ ^]^ preferentially binds more complex glycolipids bearing the Forssman antigen **9** (featuring terminal GaINAc‐α‐1,3‐GaINAc, Figure 5) which is found on sheep erythrocytes and other animals. While the galabiose moiety of GbO4 can be considered, in fact, as the minimum binding epitope, the tendency of an enhanced adhesion toward elongated saccharides is more significant for PapGIII than for PapGII.^[^ ^70^ ^]^

**Figure 6:**
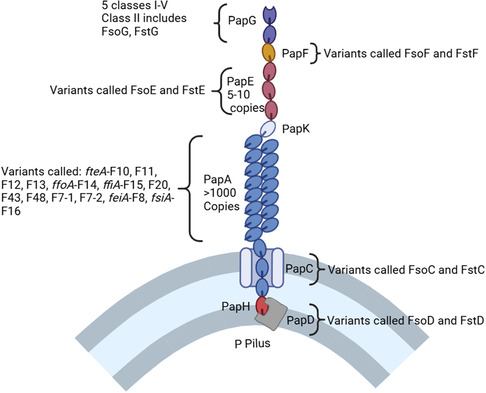
Schematic representation of structural variability of the P pilus subunits. Redrawn from^[^ ^9^ ^]^ Created in BioRender. Coyle, S. (2025) https://BioRender.com/zt95t6p.

P pili also have different major PapA subunits depending on serotype (**Figure** **6**), such as in *fteA*‐F10, F11, F12, F13, *ffoA*‐F14, *ffiA*‐F15, F20, F43, F48, F7‐1, F7‐2, *feiA*‐F8, and *fsiA*‐F16.^[^ ^71^, ^72^, ^73^ ^]^ In the F7_1_ P pilus, the FsoG (from “F‐seven‐one”) protein binds Gal‐α‐1,4‐Gal epitopes. The FsoE and FsoF proteins have been reported to be involved in the adhesion to fibronectin and basolateral membranes.^[^ ^74^ ^]^ The F7_2_ P pilus is sometimes called Fst (from “F‐seven‐two”). FstG is sometimes used as the name for the adhesin of F7_2_ P pili. Thus, FsoG and FstG correspond to PapG class II.^[^ ^75^ ^]^

F165_1_ is another type of P‐like fimbriae (similar to PapG class III/Prs) found in extraintestinal pathogenic *E. coli* (ExPEC) strains in animals and humans. F165_1_‐positive bacteria have a high stochastic phenotype switching rate (they can have ON (fimbriated), OFF (afimbriated), and also partial phases). This allows them to adapt to environmental changes during the infection cycle, which could represent increased fitness.^[^ ^76^ ^]^ F165_1_ positive *E. coli* clones show adhesion to terminal GalNAc‐α‐1,3‐GalNAc of Forssman antigen, GalNAc‐α‐1,3‐Gal, and human erythrocytes of blood group A_1_P_1_.^[^ ^77^ ^]^

A newly identified group of Pap‐like fimbriae (Plf) in EXPEC mediated adherence to host cells and colonization of the host kidney cells. Two predominant adhesin classes (PlfGI, PlfGII) were identified out of five distinct classes, according to sequence differences in the PlfG adhesin. These proteins caused hemagglutination of turkey or human erythrocytes. Interestingly, hemagglutination was not inhibited by globoside glycolipids GbO3, GbO4, or GbO5, which suggest that PlfG adhesins bind different receptors from those recognized by P fimbrial adhesin classes.^[^ ^78^ ^]^

### 2.3 S‐Fimbriae

Fimbriae of the S‐fimbrial family are frequently expressed in extraintestinal *E. coli* strains. S‐fimbriae adhesins have similar sequence identities, and the pili are organized similarly. However, their receptor specificities are different.^[^ ^79^ ^]^

**Figure 7:**
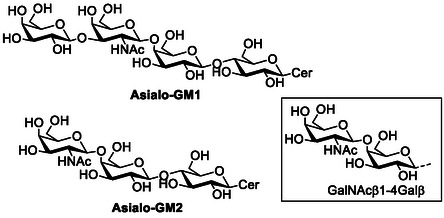
Chemical structure of asialo‐GM1 (Gal‐β‐1,3‐Gal*N*Ac‐β‐1,4‐Gal‐β‐1,4‐Glc‐Cer) and asialo‐GM2 (Gal*N*Ac‐β‐1,4‐Gal‐β‐1,4‐Glc‐Cer) ligands for FocH and disaccharide Gal*N*.Ac‐β‐1,4‐Gal‐β, all ligands for FocH.^[^ ^81^ ^]^

FocH is the fimbrial adhesin of F1C fimbriae, which are part of the S fimbria superfamily. F1C fimbriae are found on 14% to 30% of ExPEC strains of UTI origin and mediate binding to epithelial cells in the kidneys, ureters, and bladder.^[^ ^80^ ^]^ FocH has affinity for nonsialylated glycolipids such as asialo‐GM1 and asialo‐GM2; notably, the disaccharide sequence GalNAc‐β1,4‐Gal‐β, found in these glycolipids, is the high‐affinity binding epitope for the UPEC F1C fimbriae (**Figure** **7**).^[^ ^81^ ^]^ FocH has a *K*_D_ = 109 nM for asialo‐GM1, as determined by SPR analysis.^[^ ^80^ ^]^

**Figure 8:**
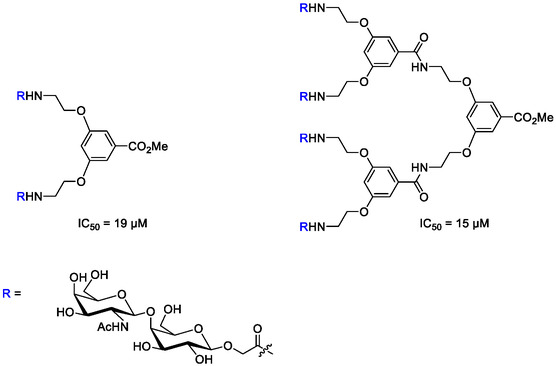
Chemical structures of selected synthetic ligands for FocH, the adhesin of FIC fimbriae, featuring binding epitope Gal*N*.Ac‐β‐1,4‐Gal.^[^ ^82^ ^]^

F1C fimbriae are also found in the *Pseudomonas aeruginosa* strains PAO and PAK. Synthetic ligands against F1C have been reported, featuring terminal Gal*N*Ac‐β‐1,4‐Gal moieties. Mono and multivalent dendritic ligands, with different spacer lengths, were investigated as inhibitors of the adhesion of *P. aeruginosa* and UPEC. The lowest IC_50_ for inhibition of binding of asialo‐GM1 was found to be 15 μM in an ELISA assay for the tetravalent derivative, only slightly lower than the IC_50_ value of 19 μM obtained for the corresponding divalent derivative (**Figure** **8**). Interestingly, comparable results were obtained against *P. aeruginosa,* despite the differences between the two pathogen*s.* ^[^ ^82^ ^]^

SfaS is the sialic acid‐specific adhesin on certain S‐fimbriae. It is expressed by *E. coli* strains causing sepsis, neonatal meningitis, and UTIs. This adhesin recognizes Neu5Ac‐α‐2,3‐Lac‐containing glycans.^[^ ^80^, ^83^ ^]^ There are two Sfa fimbriae classes, Sfa‐I and Sfa‐II (found in newborn meningitis) which differ in their major subunits and two minor subunits; however, the genes coding for their SfaS adhesins are identical.^[^ ^84^ ^]^ SfaS has been considered a virulence factor; SfaS encoding genes were found in 1.8% of ExPEC samples analyzed in a study by Lindstedt et al.^[^ ^85^ ^]^

S/F1C‐related fimbriae (Sfr) are found on *E. coli* expressing FimH.^[^ ^86^ ^]^ Sfr fimbriae are genetically homologous to Sfa.^[^ ^48^ ^]^ However, despite its homology to known lectins, its receptor specificity differs from S‐type lectins; it causes a lower amount of agglutination than Sfa when subject to anti‐Sfa serum and it does not agglutinate bovine erythrocytes (unlike Sfa).^[^ ^86^ ^]^

The analysis of the *fac* (fimbria of avian *E. coli*) gene cluster showed it is highly homologs to other S‐fimbriae gene clusters (Sfr, Sfa, and Foc). One gene is homologous with SfaAII, FacG, and FacS are homologous to SfaG‐I and SfaS‐I, while FacH is homologous to FocH.^[^ ^84^ ^]^ Other S‐type fimbriae related to FocH include F165_2_^[^ ^87^ ^]^ and CS18 fimbriae^[^ ^88^ ^]^ (both from the *fot* gene cluster). Dobrindt et al. reported a possible adhesin related to S‐type adhesins that could not be identified as any of the other adhesins grouped in the S adhesin family. It was designated Sfx.^[^ ^83^ ^]^

Other fimbrial adhesins related to S‐fimbriae include FasG, which is a subunit of 987 P(F6) fimbriae of ETEC which mediate attachment to intestinal epithelial cells.^[^ ^89^ ^]^ FasG was shown to bind galactosylceramide containing hydroxylated fatty acids and sulfatides.^[^ ^90^ ^]^ Site‐directed mutagenesis of FasG showed that the lysine residue 117 was essential for FasG‐sulfatide interaction, possibly through hydrogen bonding and/or salt bridge formation.^[^ ^91^ ^]^ Inhibiting the major subunit of 987 P‐fimbriae (FasA) with antibodies can inhibit binding to a porcine hydroxylated ceramide receptor.^[^ ^89^ ^]^ CS18 fimbriae (also known as Fot or PCFO20), found in human ETEC, are similar to 987P.^[^ ^88^ ^]^ F1B fimbrial adhesins have also been reported on *E. coli* and have similarity in their first 33 residues with F165_2_.^[^ ^92^ ^]^ Like F1C fimbriae, they have also been reported to be similar to Type 1 fimbriae.^[^ ^39^, ^86^ ^]^ CS30 isolates have also been shown to be related to 987P fimbriae, specifically to the major subunit FasA.^[^ ^93^ ^]^

### 2.4 F17 Fimbriae, GafD, UclD

The F17 fimbriae are a group of fimbriae that contain lectin domains. They have also been called G fimbriae.^[^ ^94^ ^]^ F17G adhesins present six natural variants (F17a‐fG), with F17cG also called GafD.^[^ ^95^ ^]^ Despite a lack of sequence similarity, GafD is more structurally related to FimH than PapG, though it shares common motifs with both lectins.^[^ ^96^ ^]^ F17 fimbriae types have also been referred to as Fy and Att25.^[^ ^97^ ^]^

The carbohydrate‐binding specificity of the F17G adhesins has been studied by Lonardi et al.^[^ ^95^ ^]^ F17G lectins were found to selectively recognize glycans with a terminal GlcNAc moiety, such as found in intestinal mucins. All F17G variants specifically recognize the GlcNAc‐β‐1,3‐Gal epitope, with a *K*_D_ of 0.28 mM for F17eG, as determined by SPR. F17G adhesins can bind β‐GlcNAc, with lower affinity (*K*_D_ = 1.2 mM by SPR).

**Figure 9:**
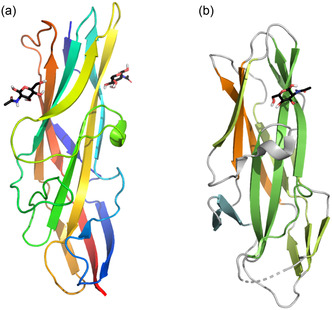
a) Graphical representation of the GafD1‐178 monomer showing both GlcNAcs bound from X‐ray structure PDB ID: 1oio. Redrawn from^[^ ^96^ ^]^ using PDB ID: [1oio] created with Flare from Cresset.^[^ ^159^, ^160^, ^161^, ^162^ ^]^ b) Graphical representation of the F17fG lectin domain with GlcNAc bound from X‐ray structure PDB ID: 1zk5. Redrawn from^[^ ^163^ ^]^ using PDB ID: [1zk5] created with the open‐source PyMOL Molecular Graphics System, Version 3.1, Schrödinger, LLC.

**Figure 10:**
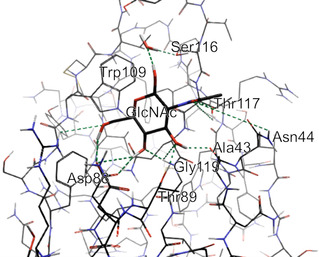
Graphical representation of GlcNAc in the binding site GafD with hydrogen bonds shown in blue from X‐ray structure PDB ID: 1oio. Redrawn from^[^ ^96^ ^]^ using PDB ID: [1oio] created with Flare from Cresset.^[^ ^159^, ^160^, ^161^, ^162^ ^]^

GafD/F17cG and F17fG were crystallized bound to GlcNAc (Table 2).^[^ ^96^, ^98^ ^]^ The crystal structures show that the monosaccharide binding site is on the side of the adhesins (**Figure** **9**). For GafD, the binding is mediated by side‐chain as well as main‐chain H‐bonding interactions. The specificity for GlcNAc arises from the arrangement around Thr117‐Asn44, where Thr117 accepts an H‐bond from the nitrogen atom in the acetamide and donates one to the carbonyl group. Trp109 provides hydrophobic interactions so that the indole ring is parallel with the plane of the sugar ring (**Figure** **10**).^[^ ^96^ ^]^

**Figure 11:**
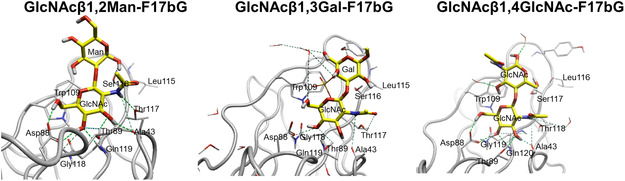
Graphical representations of GlcNAc‐β‐1,2‐Man (PDB ID: 3ffo) (left), GlcNAc‐β1,‐3 Gal (PDB ID: 4k0o) (middle), and GlcNAc‐β‐1,4‐GlcNAc (PDB ID: 2bs7) (right) in the binding site of F17bG. Redrawn from^[^ ^95^ ^]^ using PDB ID: [3ffo, 4k0o, 2bs7] created with Flare from Cresset.^[^ ^159^, ^160^, ^161^, ^162^ ^]^

The crystal structure of F17bG cocrystallized with disaccharides featuring a terminal GlcNAc (i.e., GlcNAc‐β‐1,2‐Man, GlcNAc‐β‐1,3‐Gal, and GlcNAc‐β‐1,4‐GlcNAc), shows that terminal, nonreducing GlcNAc occupies the primary binding pocket, while the other carbohydrate in the disaccharide ligand is involved in additional stacking onto the hydrophobic region neighboring the pocket (**Figure** **11**).^[^ ^95^ ^]^

**Figure 12:**
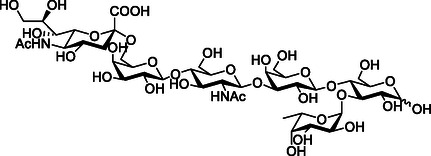
Sialyllacto‐*N*‐fucopentose VI.

UclD is a glycan binding adhesin at the tip of Ucl (F17‐like) fimbriae on ExPEC, which is homologous to GafD. Ucl fimbriae consist of four proteins, UclA‐D. A recent study by Hancock et al. on glycan binding specificity of Ucl fimbriae showed that UclD binds with the strongest affinity to sialyllacto‐*N*‐fucopentose VI ((Neu5Ac‐α‐2,6‐Gal‐β‐1,4‐GlcNAc‐β‐1,3‐Gal‐β‐1,4‐(Fuc‐α‐1,3)‐Glc), a structure possibly expressed on the gut epithelium, (**Figure** **12**) with a *K*_D_ of 11.72 nM, as determined by SPR. Comparison of the carbohydrate‐binding patterns of the UclD adhesin and UcaD (the tip adhesin from *Proteus mirabilis,* with a homologous tertiary structure) using glycan array analysis, showed that although both adhesins bind to sialyllacto‐*N*‐fucopentose VI, they recognize different glycan oligosaccharides despite their high amino acid sequence identity. Although a crystal structure of UclD with any ligand is not available, crystal structures of UcaD in complex with l‐fucose (Fuc), glucose, and galactose show a broad‐specificity carbohydrate‐binding pocket.^[^ ^99^ ^]^

### 2.5 FaeG

FaeG is the adhesin subunit on F4 fimbriae found on ETEC which binds glycolipids and glycoproteins.^[^ ^100^ ^]^ F4 fimbriae have also been called K88,^[^ ^101^ ^]^ and their binding specificity has been studied extensively in porcine pathogens. FaeG is highly conserved between different F4 serotypes (found in 88%‐100% of serotypes).^[^ ^102^ ^]^ Three main naturally occurring variants of F4 fimbriae with differences in FaeG units exist (F4_ab_, F4_ac_, and F4_ad_), each having a related, yet distinct, carbohydrate‐binding profile, which leads to different F4 receptor specificities.^[^ ^103^ ^]^ FaeG_ab_ and FaeG_ac_ bind an intestinal mucin‐type Neu5Acglycoprotein, while only FaeG_ab_ binds a porcine intestinal glycosylated transferrin.^[^ ^104^, ^105^ ^]^

**Figure 13:**
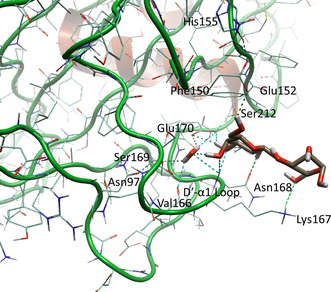
Graphical Representation of Crystal Lactose in the binding site of FaeG (F4_ad_) from X‐ray structure PDB ID: 4wei. Redrawn from^[^ ^103^ ^]^ using PDB ID: [4wei] created with Flare from Cresset.^[^ ^159^, ^160^, ^161^, ^162^ ^]^

FaeG_ad_ binds neutral glycosphingolipids, proposed to be lactosylceramide, gangliotriaosylceramide, gangliotetraosylceramide, globotriaosylceramide, lactotetraosylceramide, and lactotetraosylceramide.^[^ ^103^, ^106^ ^]^ The F4_ab_ and F4_ac_ variants show more similarities in their glycosphingolipid recognition patterns compared to the F4_ad_ variant. F4_ab_ and F4_ac_ fimbriae interacted with both sulfatide and galactosylceramide, whereas F4_ad_ fimbriae did not, binding instead gangliotriaosyl‐ and gangliotretraosylceramide.^[^ ^107^ ^]^ Nonreducing, β‐linked galactose and/or *N*‐acetylgalactosamine residues play important roles in the binding of all the fimbriae variants; Gal‐α1,3‐Gal disaccharides^[^ ^108^ ^]^ and Gal‐β‐1,3‐GalNAc and Fuc‐α‐1,2‐Gal‐β‐1,3/4‐GlcNAc^[^ ^109^ ^]^ have also identified as recognition motifs. The X‐ray structure of FaeG_ad_ bound to lactose provides a structural insight into the receptor specificity and mode of binding of the F4 fimbriae.^[^ ^103^ ^]^ Lactose interacts at the side of this FaeG, where the carbohydrate‐binding site is in a shallow groove. The interactions of the terminal galactose residue involve two short amino acid stretches, Phe150–Glu152 and Val166–Glu170, with the galactose sandwiched between the side chains of Phe150 and Lys167 (**Figure** **13**). The presence of an aromatic residue facing the nonpolar carbohydrate surface is commonly found in galactose‐binding proteins.^[^ ^103^ ^]^ Pili with the FaeG adhesin are also expressed by *Salmonella* species.^[^ ^15^ ^]^

### 2.6 F18‐Fimbriated *E. coli* (FedF)

**Figure 14:**
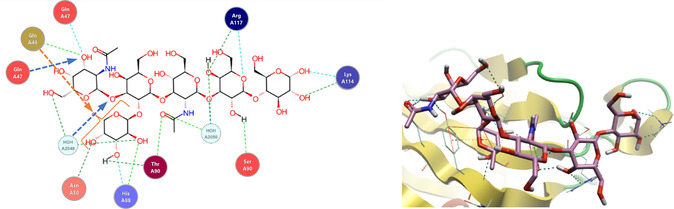
2D (left) and 3D (right) graphical representations of the blood group A type 1 hexasaccharide in the binding site of FedF from X‐ray structure PDB ID: 4b4q. In the 2D representation, interactions found by Cresset Flare from the PDB structure are shown as dashed lines representing hydrogen bonds. Arrows are interactions found only by;^[^ ^113^ ^]^ blue arrows are hydrogen bonds while orange arrows are hydrophobic interactions. Redrawn from^[^ ^111^ ^]^ using PDB ID: [4b4q] created with Flare from Cresset.^[^ ^159^, ^160^, ^161^, ^162^ ^]^

F18 pili are expressed on ETEC and Shiga toxin producing *E. coli* (STEC) in pigs. The major subunit of the pilus is FedA, with two antigenic variants named F18ac (also referred to as 8813, 2134P or Av24) and F18ab (also called F107).^[^ ^97^, ^110^ ^]^ The F18 fimbrial subtype was significantly associated with the pathogenicity of these strains, with 73.2% of the STEC isolates having F18ab genes, and 93.6% of the STEC/ETEC isolates were F18ac positive.^[^ ^110^ ^]^ FedF is the minor subunit of the protein, which serves as adhesin for this pilus. FedF has been shown to be highly conserved, with 90.4% homology across *E. coli* isolates from pigs.^[^ ^111^ ^]^ FedF mediates binding of F18‐fimbriated bacteria to glycosphingolipids having blood group ABH determinants. The minimal binding epitope was identified as blood group H‐type 1 determinant (Fuc‐α‐1,2‐Gal‐β‐1,3‐GlcNAc), but optimal binding epitopes were found to be the blood group B type 1 determinant [Gal‐α‐1,3‐(Fuc‐α‐1,2)‐Gal‐β‐1,3‐GlcNAc] and the blood group A type 1 determinant [GalNAc‐α‐1,3‐(Fuc‐α‐1,2)‐Gal‐β‐1,3‐GlcNAc].^[^ ^112^ ^]^ FedF has been characterized as a two‐domain adhesin, as demonstrated by experiments with a truncated form of the protein. This truncate construct bound blood group A type 1 hexose with a *K*_D_ = 35.2 μM (determined by SPR) and 1.76 μM (determined by backscattering interferometry, BSI). Crystal structures of this truncated cocrystallized with blood group B type 1 hexasaccharide (PDB ID: 4b4r) and blood group A type 1 hexasaccharide (PDB ID: 4b4q) (**Figure** **14**) have also been reported.^[^ ^113^ ^]^

Furthermore, FedF has also been shown to bind sulfated *N*‐acetyllactosamine and lactose derivatives. The best binder was determined to be Gal‐β‐1,4‐GlcNAc sulfated at positions 3′, 6′, and 6 as determined by glycan array analysis.^[^ ^95^ ^]^

Successful inhibition of FedF attachment has been achieved in piglets through the development of nanobodies. Dissociation constants for the four most effective nanobodies were determined using Microscale Thermophoresis (MST), with *K*_D_ values ranging from 29 nM to 1.58 nM.^[^ ^114^ ^]^ One of these nanobodies has also been successfully crystallized with the FedF lectin domain (PDB ID: 4w6x). Amino acid residues 60 to 109 were necessary for F18 binding to porcine epithelia, with a disulfide bridge between Cys64 and Cys83 being essential for binding.^[^ ^115^ ^]^

### 2.7 Other Chaperone‐Usher Adhesins

**Figure 15:**
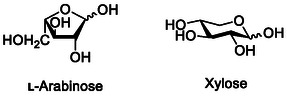
Chemical structure of l‐arabinose and xylose, recognized by ECP and Yad fimbriae, respectively.

Adhesin FanC is the major subunit of F5 fimbriae (previously called K99),^[^ ^116^ ^]^ found in porcine, bovine, and ovine ETEC.^[^ ^117^, ^118^ ^]^ F5 fimbriae bind sialylated glycolipids,^[^ ^119^ ^]^ including Neu5Ac‐α‐2,3‐Gal‐β‐1,3‐GlcNAc, as reported by Day et al. using a recombinant *E. coli* strain expressing different CU fimbriae, together with glycan array analysis.^[^ ^80^ ^]^ This study also reported that ExPEC adhesin I (Yqi adhesin), associated with adhesion and colonization of the lungs of chickens in avian‐pathogenic *E. coli* (APEC), recognized structures featuring terminal β‐GlcNAc. It also binds structures containing l‐fucose such as Lewis A, blood group antigen B, and α‐Gal‐Lewis X. The structures recognized by Yqi are widely expressed across various tissue types and host species. Blood groups and Lewis antigens are common targets for pathogens.^[^ ^80^ ^]^ In addition, Day et al.'s analysis also identified the binding of *E. coli* common pilus (ECP) fimbriae to Gal‐α‐1,6‐Glc, asialo‐GM1, blood group B trisaccharide, and GlcNAc‐β‐1,6‐Gal‐β‐1,4‐GlcNAc. The tip adhesin (EcpD) in ECP allows binding to the glycoproteins fibronectin, laminin, collagen I and IV, and mucins.^[^ ^120^ ^]^ ECP is sometimes referred to as YAG.^[^ ^15^ ^]^ Interestingly, ECP targets l‐arabinosyl residues (**Figure** **15**) with α‐1,5 links and longer arabinan chains.^[^ ^121^ ^]^ These sugars are commonly found in plant cell walls but rarely in animals.

Yad fimbriae are commonly found in UPEC. In addition to the *yad* operon, several other operons such as *ycb* (also known as *elf*, *E. coli* laminin fimbriae or *loc5*), *yfc*, *yra*, *sfm*, and *yeh* encode functional fimbriae that contribute to *E. coli*'s ability to adhere to various surfaces, including abiotic ones.^[^ ^122^, ^123^ ^]^ The *yad* operon is also referred to as *loc2*.^[^ ^123^ ^]^ Xylose (Figure 15), another sugar commonly found in plants, targets YadC and has shown effectiveness in both the prevention and treatment of UPEC infections. The receptor for YadC has been identified as annexin A2.^[^ ^124^ ^]^

AF/R1 fimbriae enable *E. coli* to adhere to rabbit small intestine epithelial cells. This binding is mediated by glycolipids (proposed to be galactosylceramides) and glycoproteins featuring sialic acid and β‐galactosyl residues.^[^ ^125^ ^]^ F42 is a colonization factor of ETEC found in piglets, but it also binds to glycoproteins and causes agglutination in chicken erythrocytes; hemagglutination caused by this adhesin may be inhibited by GalNAc.^[^ ^126^ ^]^

MrkD is expressed on type 3 fimbriae found in *E. coli* and *Klebsiella pneumoniae*. MrkD primarily binds to type V collagen^[^ ^37^, ^127^ ^]^ but some variants can also bind to mannan. MrkD structure consists of a lectin domain with a putative binding pocket and a fimbria‐anchoring pilin domain; these domains bind the target in a catch bond‐like manner, with enhanced binding under increasing shear conditions. This resembles the binding mechanism of FimH. Catch‐bonds are a type of interaction in adhesin‐receptor complexes that increases the lifetime of these complexes under tensile mechanical force, their mechanism in FimH has been described by Sauer et al.^[^ ^128^ ^]^ Interestingly, although MrkD and FimH only have 12% amino acid sequence similarity, homology modeling proposes comparable structures for these adhesins.^[^ ^129^ ^]^ MrkD is also encoded in species including *Klebsiella aerogenes*, *Enterobacter hormaechei*, *Enterobacter cloacae*, *Citrobacter freundii*, and *Citrobacter koseri*; it may also be expressed by some *Salmonella* species.^[^ ^15^, ^16^ ^]^

## 3 Class 5 Fimbrial Adhesins

Class 5 fimbriae in ETEC include eight distinct types that mediate adhesion to the small intestine. These fimbriae are divided into three subclasses: 5a (CFA/I, CS4, CS14), 5b (CS1, CS17, CS19, PCFO71), and 5c (CS2). They contain minor adhesin subunits.^[^ ^130^ ^]^

CFA/I fimbriae are composed of a major repeating subunit, CfaB, and a single tip subunit, CfaE. The tip adhesin, CfaE, of CFA/I appears to bind sialylated proteins at a binding site with three arginine residues^[^ ^131^ ^]^ while the major subunit, CfaB, binds nonsialylated glycosphingolipids.^[^ ^132^ ^]^ For CS1, the adhesin CooD also requires arginyl residues for binding and is associated with CFA/II colonization factors.^[^ ^133^ ^]^ CS2 fimbriae are also specific to sialylated glycans. Fimbrial binding to erythrocytes could be inhibited with low concentrations of sialyl‐lactose.^[^ ^134^ ^]^ The tip adhesins for CS2, CS4, CS17, and CS19 fimbriae are CotD, CsaE, CsbD, and CsdD, respectively.^[^ ^42^, ^135^, ^136^ ^]^ Both major and minor subunits of class 5 fimbriae show high homology within their subclasses.

### 3.1 Fimbriae Related to Class 5 Alternate

Class 5 fimbriae alternate include several types of adhesins that mediate adhesion in ETEC. CS6, one of the most commonly detected nonfimbrial adhesins, is composed of two subunits, CssA and CssB. The CssB subunit is responsible for highly specific binding to sulfatide (SO_3_‐3 Gal‐β‐1‐Cer),^[^ ^137^ ^]^ while the CssA subunit was found to recognize fibronectin, although this is not a carbohydrate‐mediated interaction.^[^ ^138^ ^]^

CS3 fimbriae are produced by CFA/II type ETEC. They mediate binding to glycoproteins in intestinal cell membranes featuring galactosylated glycans, with GM1, asialo‐GM1, and GM2 inhibiting this interaction. This highlights that GalNAc‐β‐1,4‐Gal is necessary for CS3 binding. This disaccharide has not only been implicated as a binding epitope for other fimbriae in EPEC but also for other pathogens like *Pseudomonas aeruginosa*.^[^ ^139^ ^]^

CS7 fimbriae belong to the CS5 group of class 5 fimbriae and weakly bind isoglobotriaosylceramide, lactosylceramide, and neolactotetraosylceramide.^[^ ^132^, ^140^ ^]^

## 4 Type IV Pili

Adhesive type IV bundle‐forming pili (BFP) are in EPEC. The pilin subunit, BfpA, has diverse alleles divided into two groups: α and β. α‐BfpA mediates adherence to host cells via *N*‐acetyllactosamine in human intestinal cells and HEp‐2 cells, but β‐BfpA does not recognize it.^[^ ^141^ ^]^

CS21 (or longus) is type IVb pili found in many ETEC. CS21's major subunit, LngA, mediates adhesion to intestinal epithelial cells through binding of sialylated glycans.^[^ ^142^ ^]^

Longus is highly related to, but distinct from, CFA/III.^[^ ^143^ ^]^ CFA/III is a type IVb pilus which has a minor pilin, CofB, with an H‐type lectin domain at its tip. However, a secreted protein CofJ, encoded within the same CFA/III operon, binds the expected carbohydrate recognition site of the CofB's H‐type lectin domain. An X‐ray crystal structure of CofB complexed with a peptide containing the binding region of CofJ and solution data were used by Oki et al. to propose a model for the CofJ–CFA/III pilus complex, necessary for binding the host cell membrane.^[^ ^144^ ^]^

## 5 Miscellaneous

Often, the terms pili and fimbriae are used interchangeably, though reserving the term pili only to pili mediating conjugation has been proposed. However, fimbrial structures are occasionally categorized into two types: short fibrils and longer flexible structures. Some consider fibrils distinct from fimbriae. Thin pili are also known as fibrillae.^[^ ^101^, ^116^, ^145^, ^146^, ^147^ ^]^ Rod‐shaped pili may transition to fibrillar structures, while curli (found in *E. coli* and *Salmonella* species) have been occasionally referred to as amyloid fibrils and fimbriae.^[^ ^32^, ^148^ ^]^ Nonfimbrial and fibrillar lectins present on *E. coli* are shown in **Table** **3**.

**Table 3:** “Nonfimbrial” and “fibrillar" lectins in *E. coli*.

Structure name	Adhesin	Ligand	Reference
CS6 CFA/IV^[^ ^135^ ^]^	CssB	SO_3_‐3‐Gal‐β‐1‐Cer^[^ ^137^ ^]^ [SIF68] SO_3_‐3‐Gal‐β‐1,4‐Glc‐β‐1‐Cer^[^ ^137^ ^]^ [SIF69]	[138]
F41 fimbriae Similarities to K88 (CU)^[^ ^173^ ^]^	F41 lectin	Acidic monosaccharides^[^ ^150^ ^]^ Erythrocyte glycophorins^[^ ^150^ ^]^ NN blood type glycophorin^[^ ^150^ ^]^	[149]
STEC Autoagglutinating adhesin (Saa)	Saa	Mannose^[^ ^151^ ^]^ [SIF70]	[151]
Lymphostatin (outer membrane)	LifA Efa‐1/Efa1^[^ ^11^, ^152^ ^]^	Recombinant lymphostatin binds uridine diphosphate GlcNAc^[^ ^152^ ^]^ [SIF71]	[152]
Presented on flagellin	EtpA	Binds blood group A antigens^[^ ^154^ ^]^ [SIF72] GalNAc, *K*_d_ ≈ 1.6 × 10^−8^ M^[^ ^154^ ^]^ [SIF4]a)	[153]
Outer membrane protein A	OmpA	GlcNAc‐β‐1,4‐GlcNAc epitopes^[^ ^155^ ^]^ [SIF28] 1,4‐linked GlcNAc oligomers^[^ ^155^ ^]^	[155]
CS31A	ClpG	GlcNAc^[^ ^156^ ^]^ [SIF73] Neu5Ac^[^ ^156^ ^]^ [SIF64] *N*,*N'*‐Diacetylchitobiose^[^ ^156^ ^]^ [SIF74] *N,N',N''*‐Triacetylchitotriose^[^ ^156^ ^]^ [SIF75]	[156]

a) IC_50_s, MICs, or *K*_d_s of ligands are given when known.

The F41 lectin of ETEC is known to bind sialic acid and the sialoglycoprotein glycophorin in erythrocytes of the MM and, less strongly, the NN blood types.^[^ ^149^, ^150^ ^]^ The STEC autoagglutinating adhesin, also found in EHEC and verotoxigenic *E. coli* (VTEC), shows adherence sensitive to mannose.^[^ ^151^ ^]^ Lymphostatin (LifA) is found in adherent EPEC and in EHEC as Efa1.^[^ ^11^ ^]^ It is involved in adherence, though its mode of action is not well understood. Recombinant lymphostatin binds uridine diphosphate *N*‐acetylglucosamine and contains a glycosyltransferase domain similar to large clostridial toxins.^[^ ^152^ ^]^ EtpA is an enterotoxin secreted and captured by the tips of flagellin for presentation to its receptors,^[^ ^153^ ^]^ which has also been reported to bind to blood group A antigens.^[^ ^154^ ^]^ OmpA is an outer membrane protein produced by K1 *E. coli* that mediates attachment to endothelial cells recognizing specifically GlcNAc‐β‐1,4‐GlcNAc and 1,4‐linked GlcNAc oligomers from chitin.^[^ ^155^ ^]^ CS31A has been found to bind GlcNAc and Neu5Ac.^[^ ^156^ ^]^ Finally, a galactose‐specific fimbrial adhesin was identified in an EAEC strain. The binding of this strain to HEp‐2 cells was inhibited by galactose. Antibodies for the adhesin were developed, but prevented galactose binding, suggesting these antibodies bound at the sugar‐binding site.^[^ ^157^ ^]^

## 6 Summary and Outlook

We have outlined some of the most significant carbohydrate‐binding adhesins (other than FimH) found in fimbria and pili of different strains of pathogenic *E. coli*. In light of the antimicrobial resistance crisis, the need for new therapeutic alternatives to treat infectious diseases brings attention back to fimbrial adhesins, with the potential to serve as a target for antivirulence therapies. The extensively investigated mannose‐based compounds targeting FimH for the treatment of UTIs highlight the potential of this approach.^[^ ^24^ ^]^ However, there are limited examples of synthetic inhibitors for other prevalent *E. coli* carbohydrate‐binding proteins (lectins) despite the wide range of adhesins that are considered virulence factors and are known to interact with host glycans to initiate infection and colonization. One major hurdle in the development of successful antagonists of fimbrial adhesins has been the limited structural knowledge of said proteins, with a relatively small number of crystallographic structures reported so far. The crystallographic structure of some known lectins can aid in the development of inhibitors; however, not all lectins have known binding modes. The study of how lectins interact with their carbohydrate receptors may enable in silico drug design of lectin inhibitors. Nevertheless, the considerable energetic contributions of water to the binding of some lectins combined with their shallow binding sites complicate the rational design of high‐affinity binding drugs through structure‐based methodologies. Traditional approaches to enhance binding affinity, such as the design of multivalent or glycomimetic compounds which are successfully being applied to other carbohydrate‐binding proteins such as galectins, have been explored only for some adhesins; these strategies to enhance binding strength are yet to be optimized in suitable adhesin targets. Moreover, new technologies such as Alphafold, which allows for rather accurate prediction of protein structure from amino acid sequence, are becoming highly valuable tools to assist in the design of new glycomimetic ligands for carbohydrate‐binding fimbrial adhesins for which crystallographic (or cryoEM) structures are not yet available. Finally, many adhesins have yet unknown receptor specificities. In this regard, advanced glycan array methodologies can be extremely useful to assess the binding of purified lectins or whole‐cell recombinant adhesins^[^ ^80^ ^]^ which can lead to the identification of new carbohydrate‐binding bacterial targets.

As discussed throughout this review, certain adhesins with defined binding specificities are more frequently found in some serotypes, especially within defined pathotypes. A single serotype can express multiple adhesins, and different strains with the same serotype can have different adhesion profiles. Moreover, these adhesins play a key role in determining the tissue tropism and virulence of each group. This reflects the functional complexity of host‐pathogen interactions and adaptation strategies in *E. coli.* For example, FimH found in UPEC binds mannose‐containing glycoproteins on bladder epithelial cells, but glucoside binding PapG adhesins are also commonly found in pyelonephritis‐associated UPEC strains. In addition, PapG adhesins have been identified in several *E. coli* strains causing neonatal meningitis.^[^ ^158^ ^]^ This complexity can be harnessed towards the development of innovative therapeutics and diagnostic tools to treat and diagnose *E. coli* infections. In addition to the examples already discussed earlier in this review, which consider mainly FimH, FmlH, and PapGII, antiadhesion therapies targeting other well structurally characterized adhesins can be investigated to block pathogen binding using antibodies or synthetic molecules which mimic host cell receptors, binding to adhesins and preventing pathogens from attaching to host tissues. Similarly, these recognition moieties can also be exploited in targeted drug delivery applications, whereby they can be incorporated onto nanoparticles or drug carriers to target the pathogen specifically. Additionally, *E. coli* adhesins could be engineered to target specific tissues or cells, improving drug localization and reducing side effects. The remarkable achievements obtained so far with FimH antagonists and other ligands targeting carbohydrate‐binding proteins pave the way for exciting developments in this field of research.

## Conflict of Interest

The authors declare no conflict of interest.

## Supporting information

Supplementary Material
