# Supplementary material for: Beyond FimH: Diversity and Relevance of Carbohydrate‐Binding Fimbrial Proteins in Escherichia coli
Source: Chembiochem. 2025 Jul 30;26(17):e202500433. doi: 10.1002/cbic.202500433 (PMC12442223; doi:10.1002/cbic.202500433)
Supplement: Supplementary file 1 — Supplementary Material [file CBIC-26-e202500433-s001.pdf]

Supporting Information

Beyond FimH: Diversity and Relevance of Carbohydrate-Binding Fimbrial Proteins in *Escherichia coli*

Oliwier R. Dulawa,[a, c] Shane M. Coyle,[a, c] Fiona Walsh,*[b, c] and Trinidad Velasco-Torrijos*[a, c]

Table of contents

1. Graphical representations of glycans from **Table 1** and **Table 3**.

1. Graphical representations of glycans from Table 1 and Table 3.

Glycans are represented as chemical structure and using the Symbol Nomenclature for Glycans (SNFG).

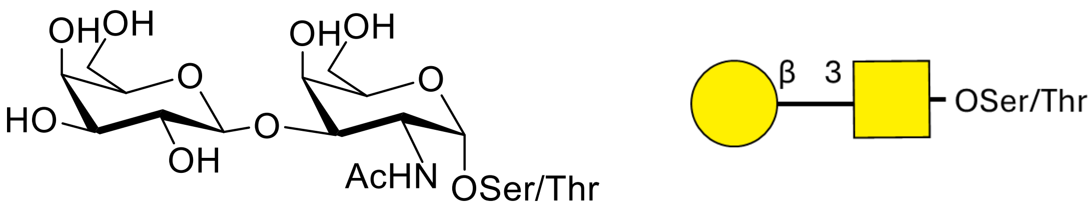

Figure S1. The TF antigen

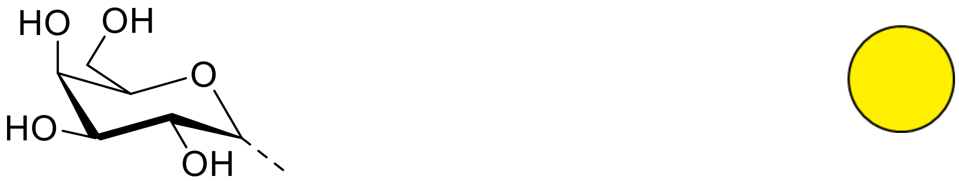

Figure S2. Terminal galactose

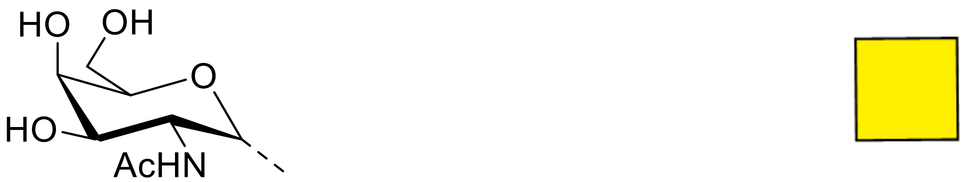

Figure S3. Terminal *N*-acetylgalactosamine

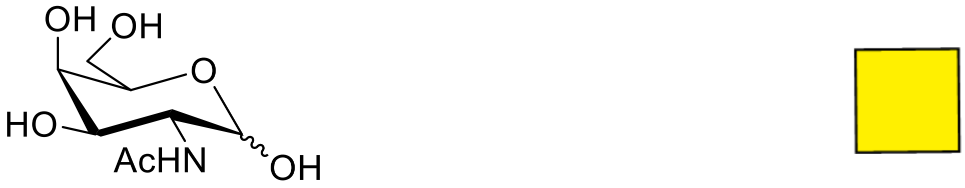

Figure S4. *N*-Acetylgalactosamine

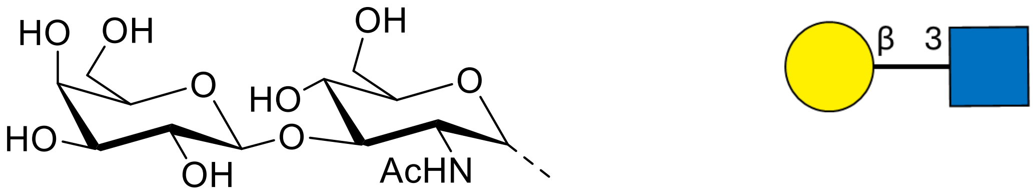

Figure S5. Terminal Gal-β-1,3-GlcNAc

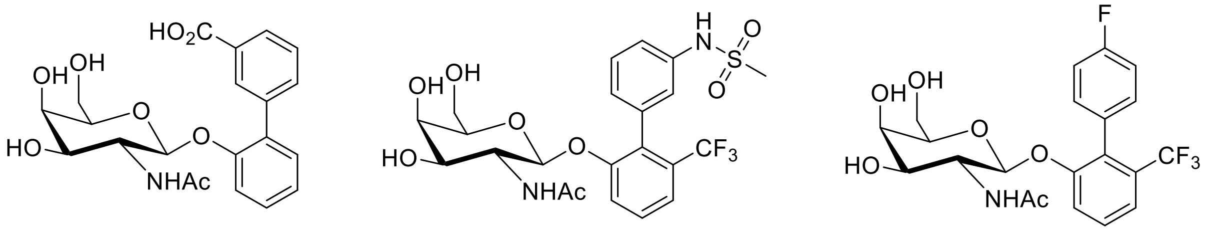

IC_{50} = 0.64 μM

IC_{50} = 0.034 μM

IC_{50} = 0.19 μM

Figure S6. Synthetic ligands for FmlH

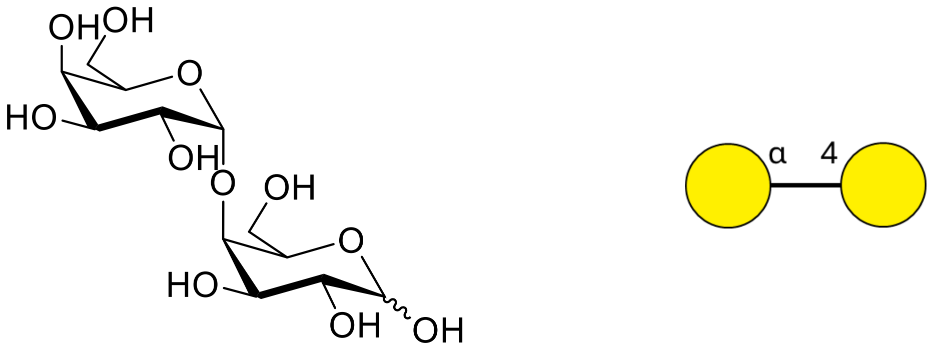

Figure S7. Gal-α-1,4-Gal

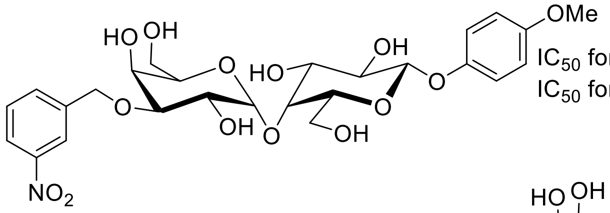

IC$_{50}$ for PapGI = 4.1 µM;
IC$_{50}$ for PapGII = 180 µM.

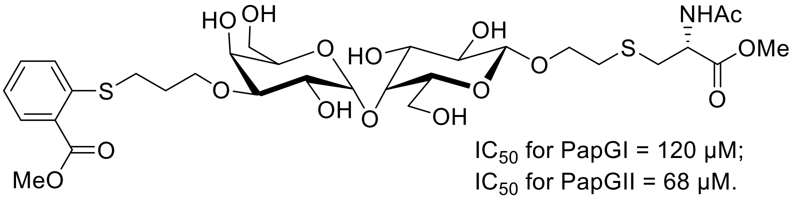

IC$_{50}$ for PapGI = 120 µM;
IC$_{50}$ for PapGII = 68 µM.

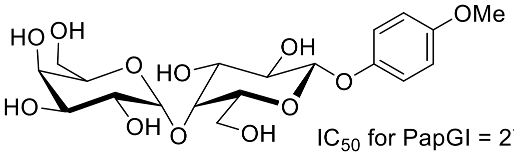

IC$_{50}$ for PapGI = 270 µM;
IC$_{50}$ for PapGII = 470 µM.

IC$_{50}$ for PapGII = 2 µM

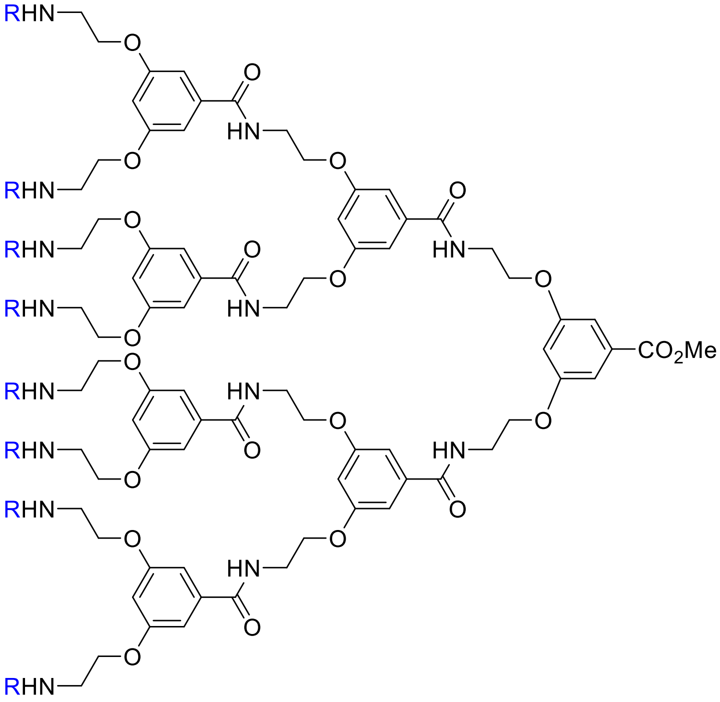

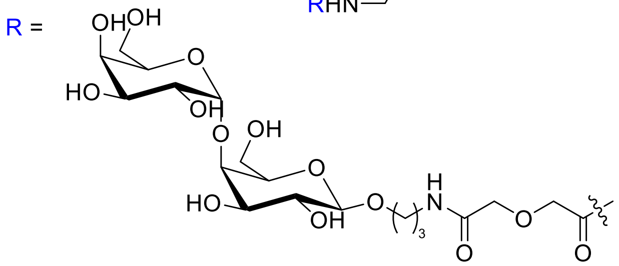

Figure S8. Synthetic ligands for PapGI and PapGII

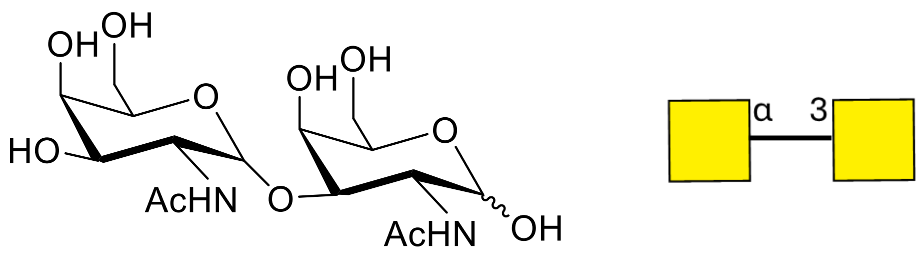

Figure S9. GalNAc-α-1,3-GalNAc

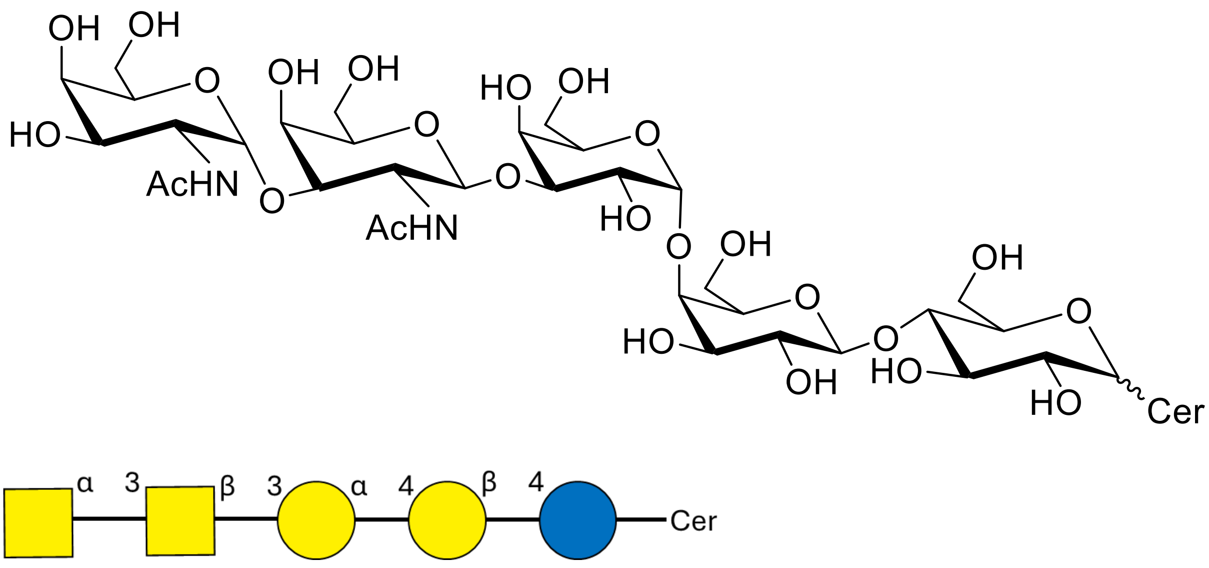

Figure S10. Globoside-5

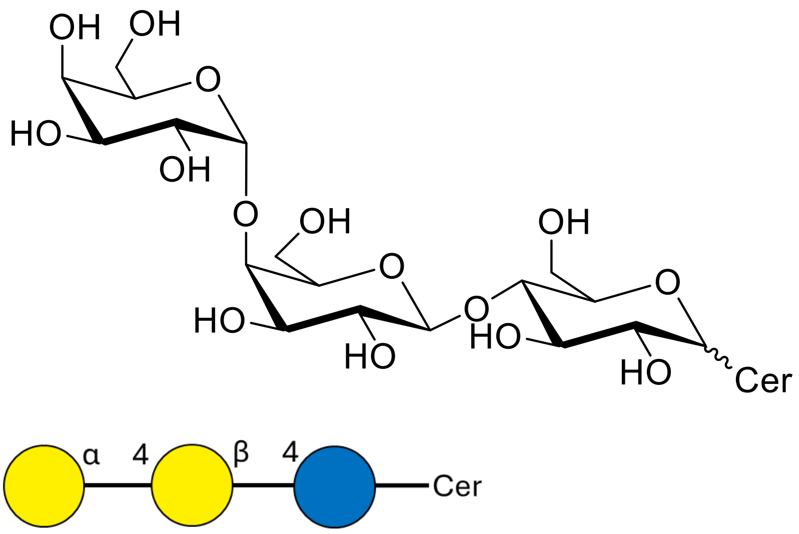

Figure S11. Globoside-3

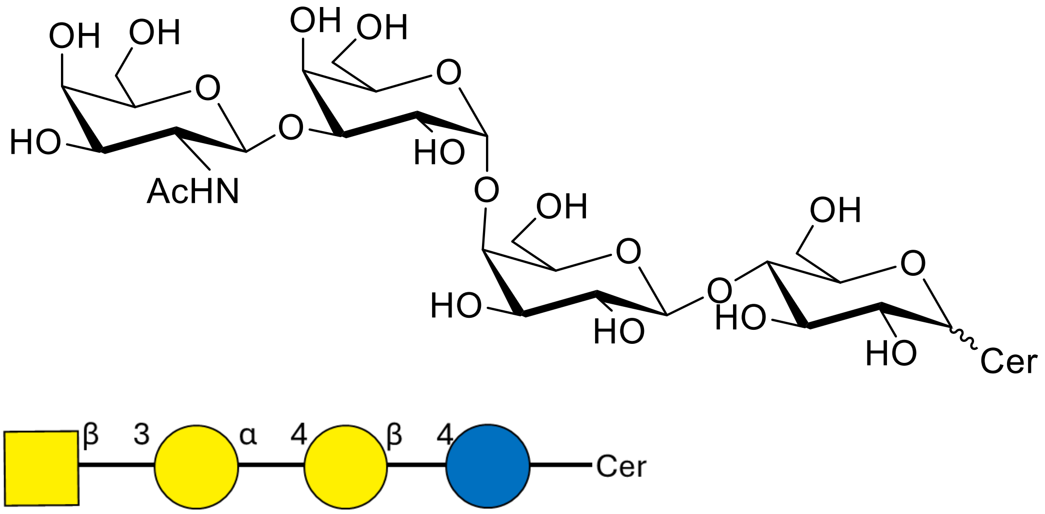

Figure S12. Globoside-4

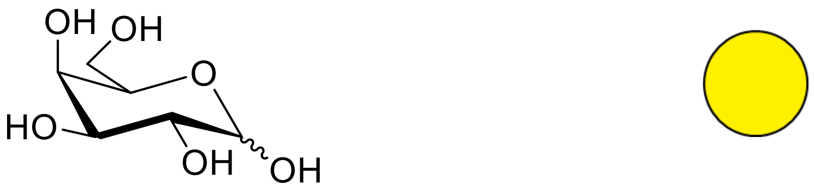

Figure S13. Galactose

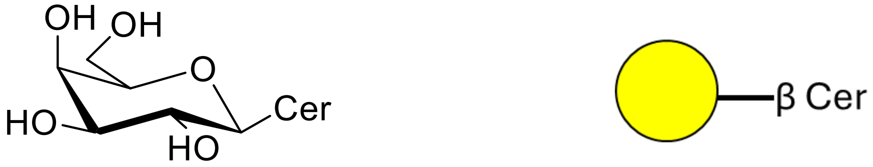

Figure S14. Gal-β-1-Cer

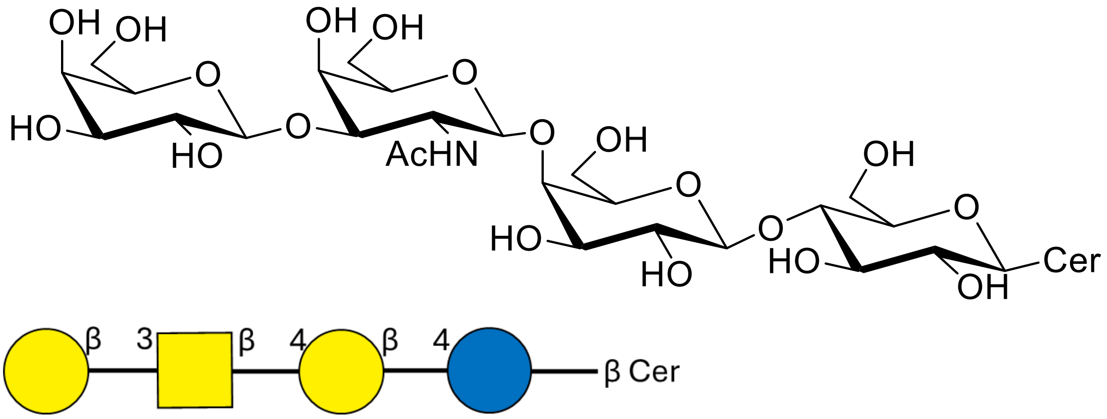

Figure S15. Asialo-GM1

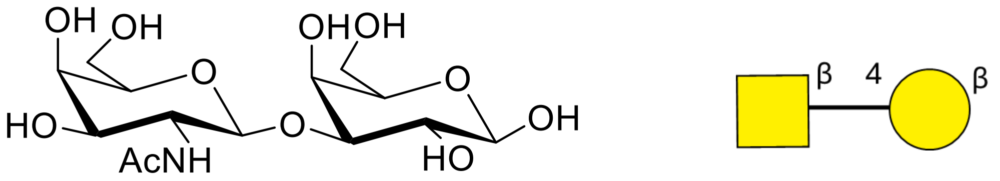

Figure S16. GalNAc-β-1,4-Gal-β

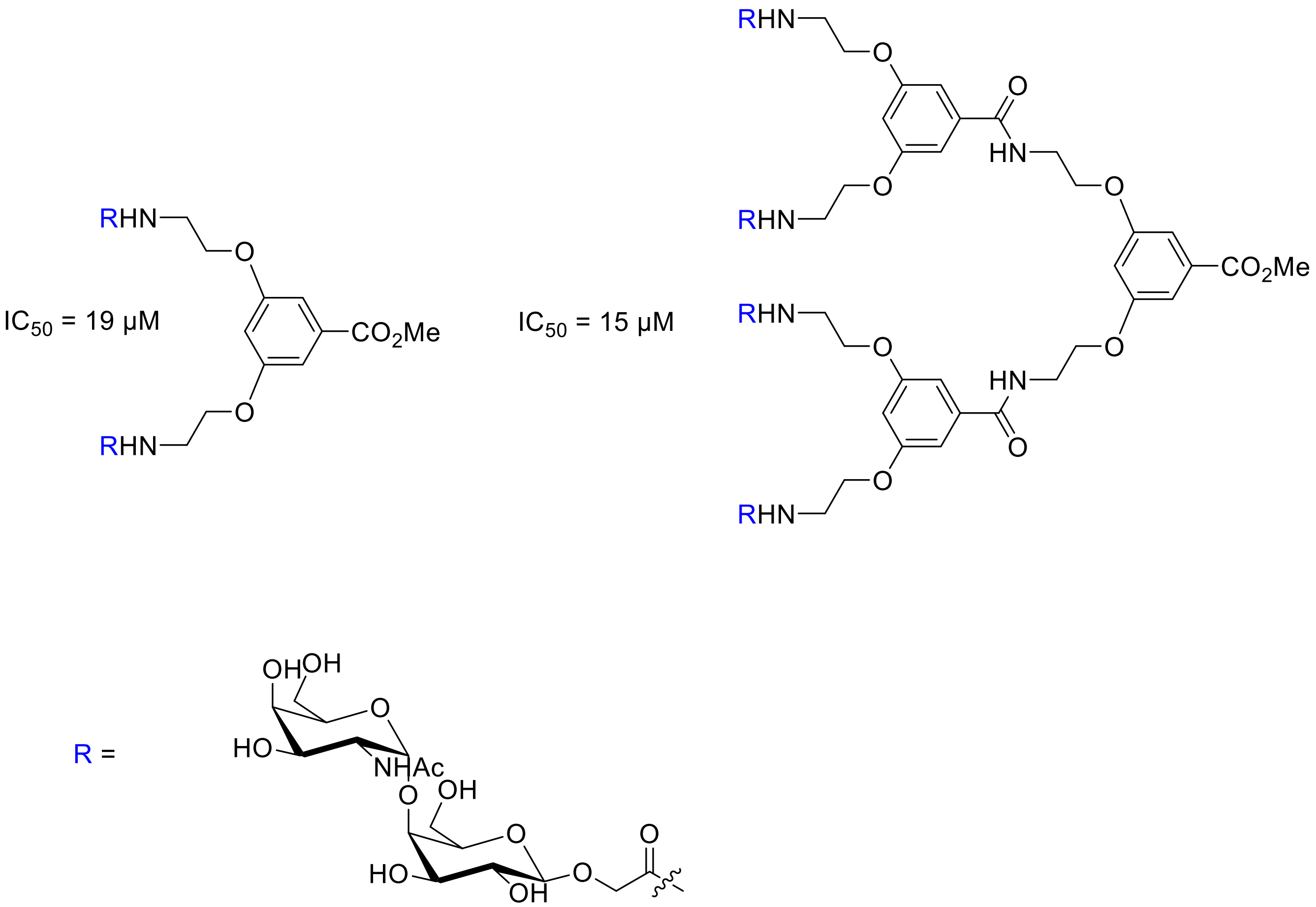

Figure S17. Synthetic ligands for FocH

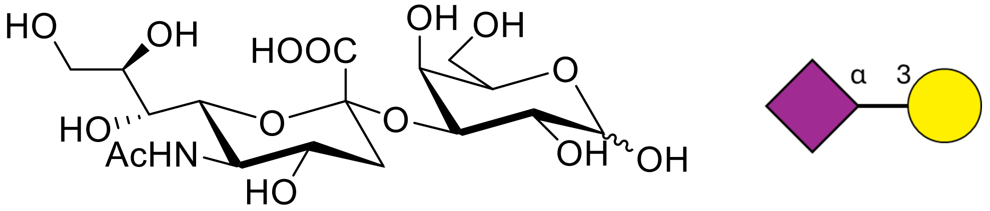

Figure S18. Neu5Ac-α-2,3-Gal

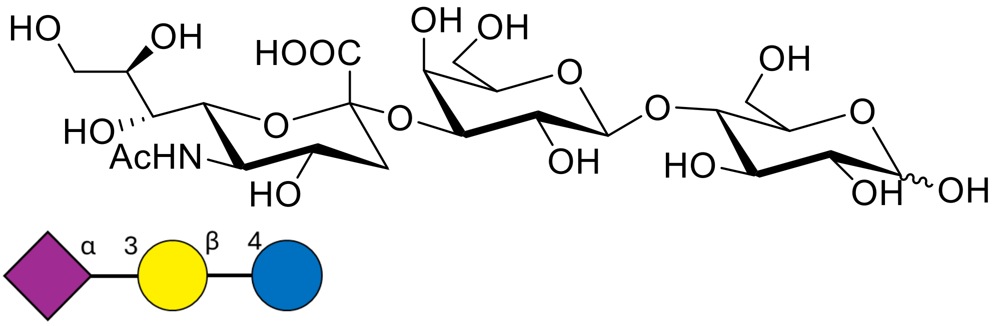

Figure S19. Neu5Ac-α-2,3-Lac

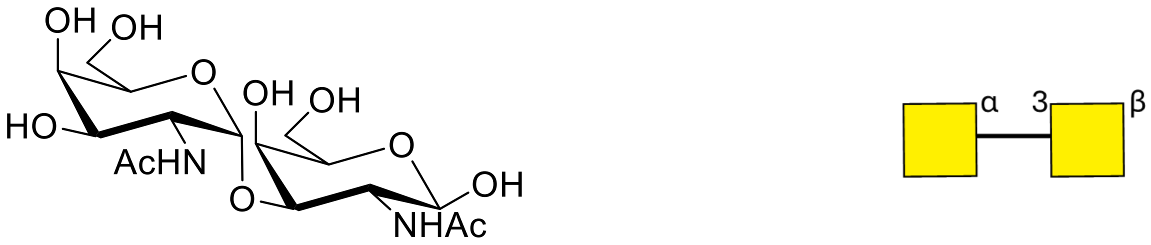

Figure S20. GalNAc-α-1,3-GalNAc-β

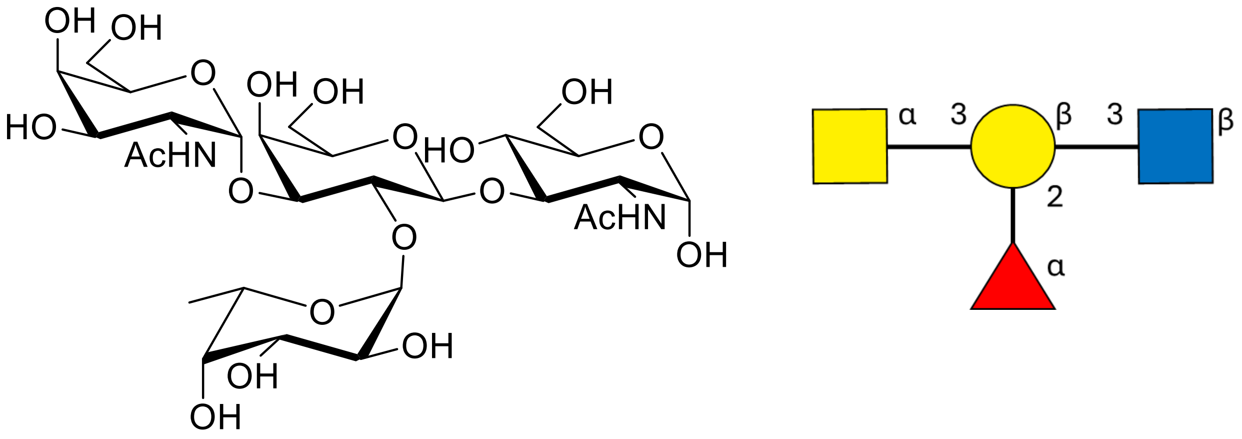

Figure S21. Blood group antigen A_1

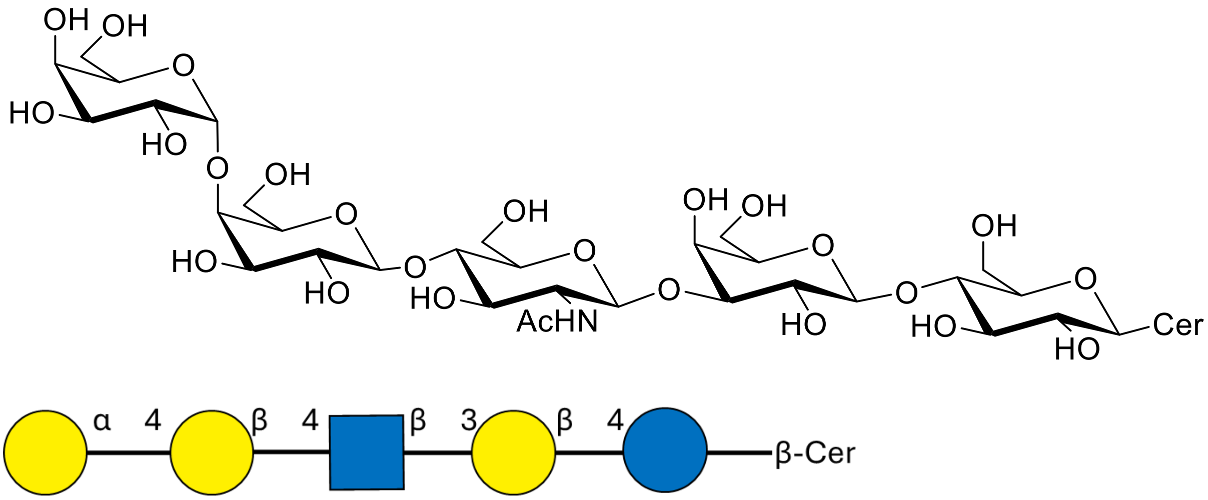

Figure S22. Blood group antigen P_1

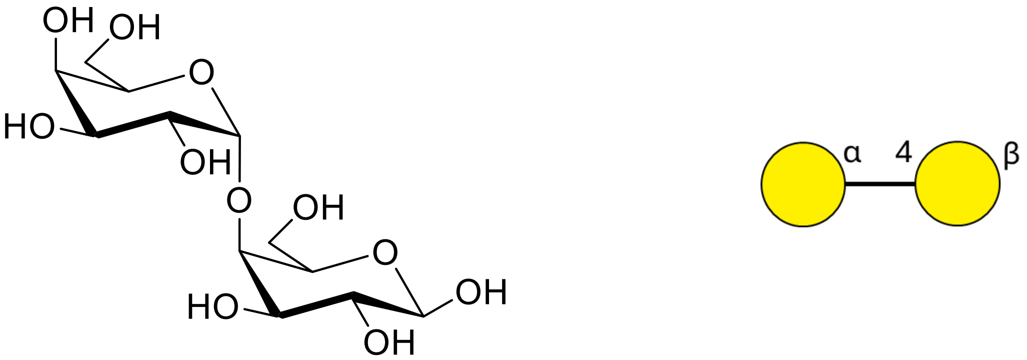

Figure S23. Gal-α-1,4-Gal-β

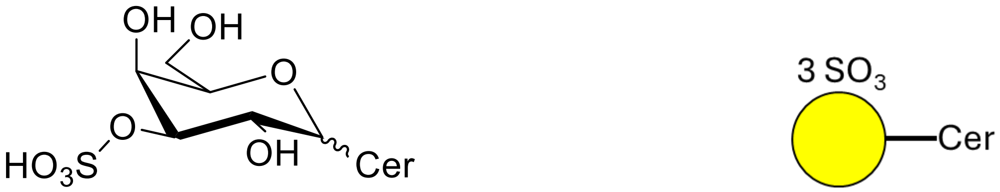

Figure S24. Sulfatide

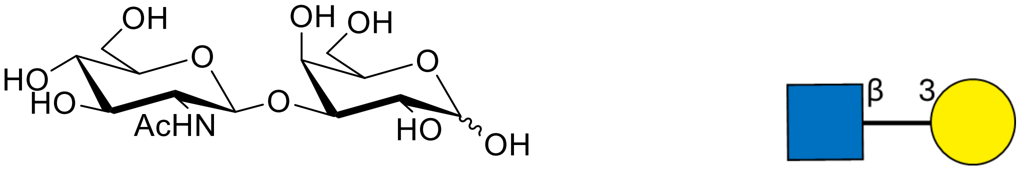

Figure S25. GlcNAc-β-1,3-Gal

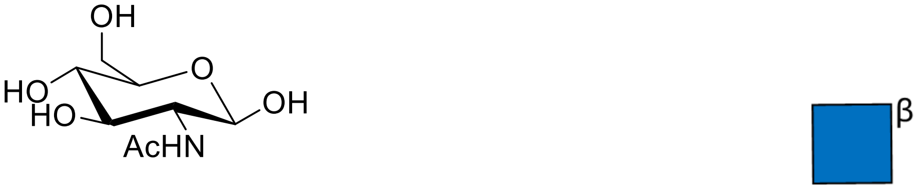

Figure S26. β-*N*-Acetylgalactosamine

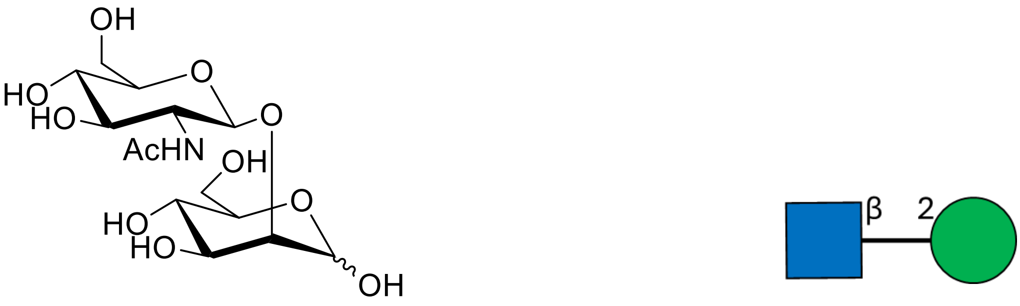

Figure S27. GlcNAc-β-1,2-Man

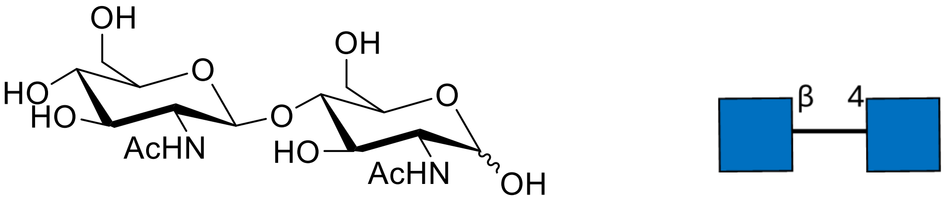

Figure S28. GlcNAc-β-1,4-GlcNAc

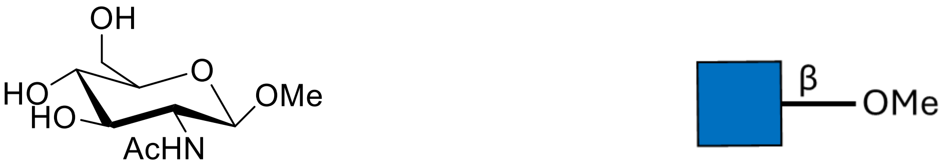

Figure S29. GlcNAc-β-1-OMe

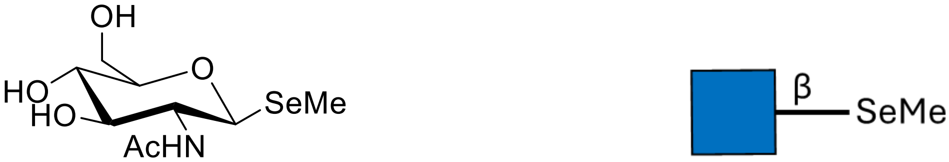

Figure S30. GlcNAc-β-1-SeMe

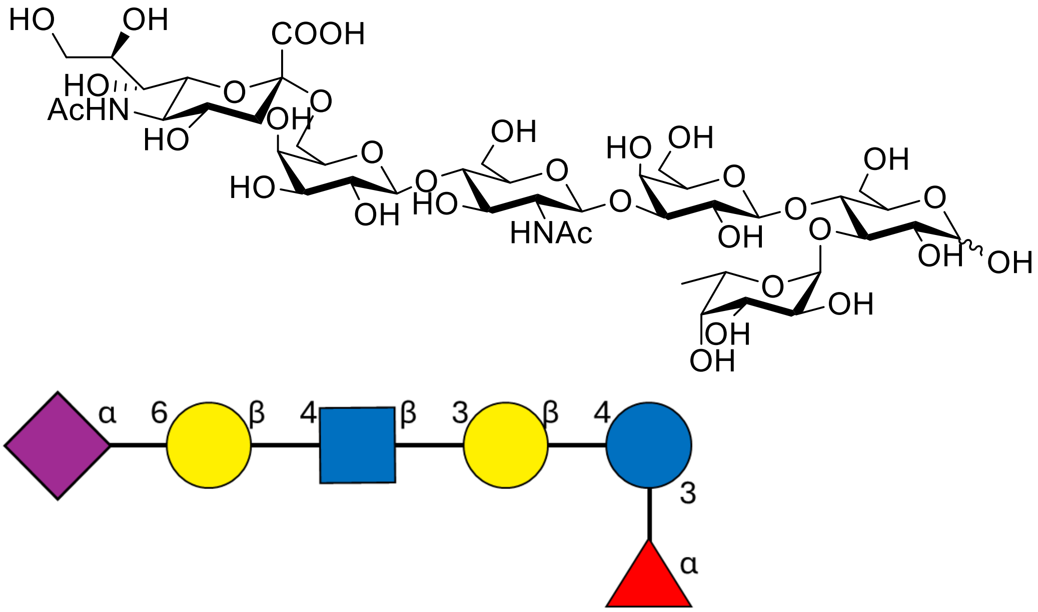

Figure S31. Sialyllacto-*N*-fucopentose VI

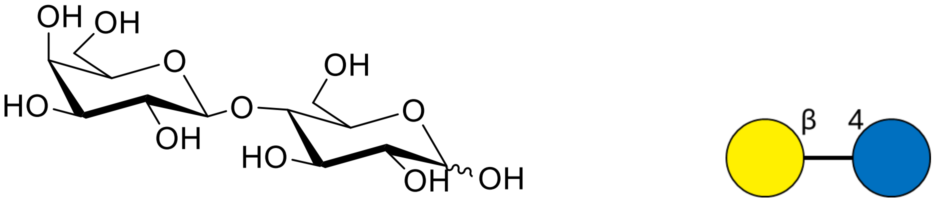

Figure S32. Lactose

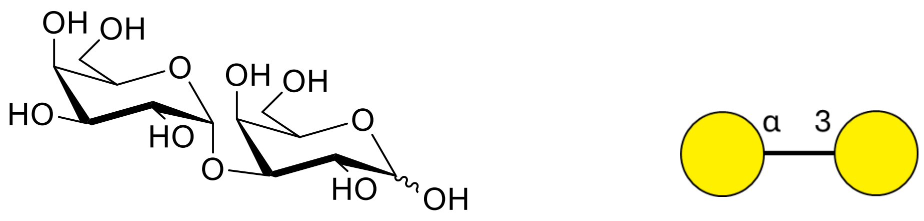

Figure S33. Gal-α-1,3-Gal

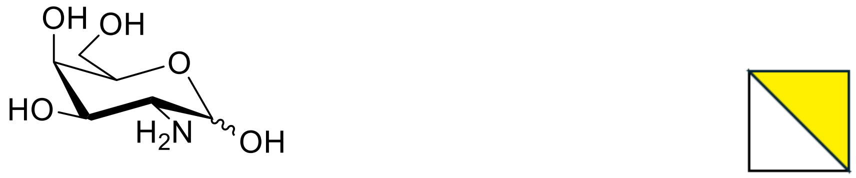

Figure S34. Galactosamine

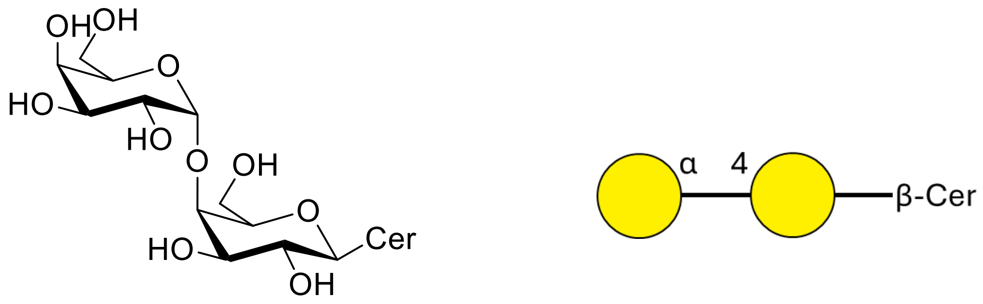

Figure S35. Gal-α-1,4-Gal-β-1-Cer

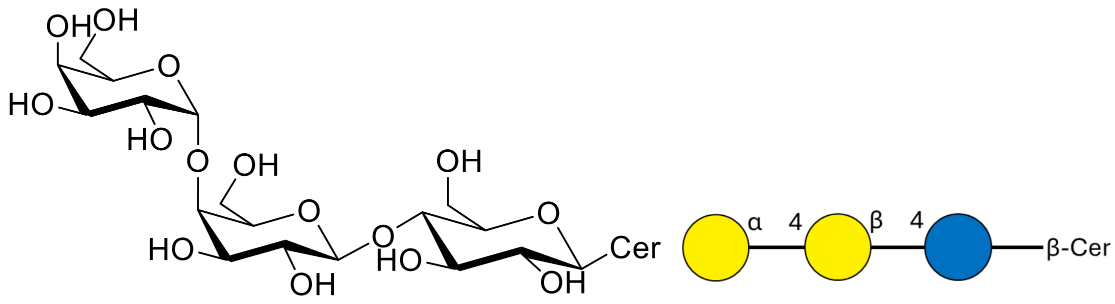

Figure S36. Gal-α-1,4-Gal-β-1,4-Glc-β-1-Cer

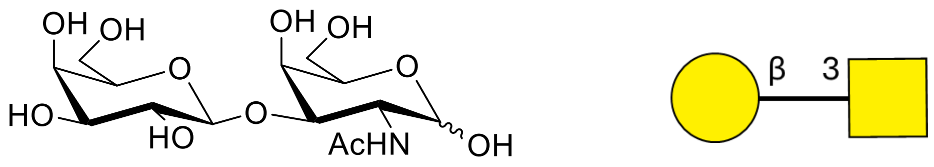

Figure S37. Gal-β-1,3-GalNAc

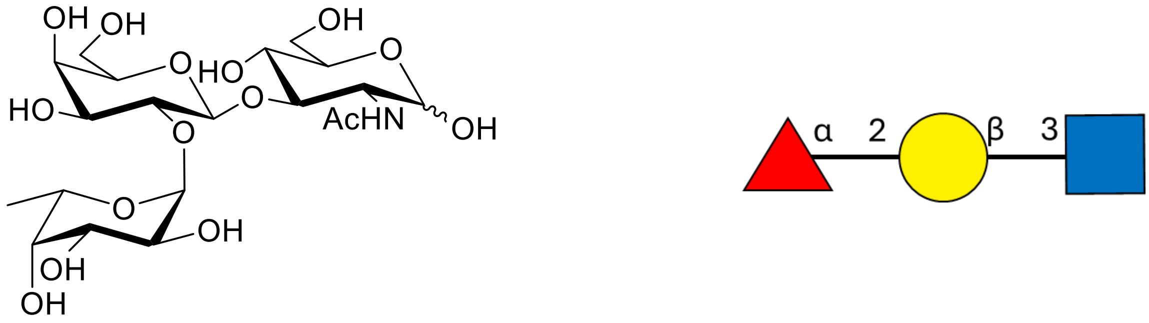

Figure S38. Fuc-α-1,2-Gal-β-1,3-GlcNAc

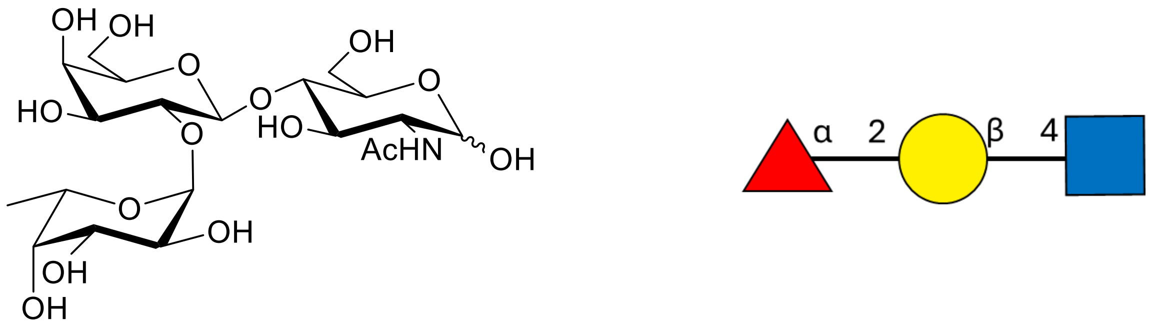

Figure S39. Fuc-α-1,2-Gal-β-1,4-GlcNAc

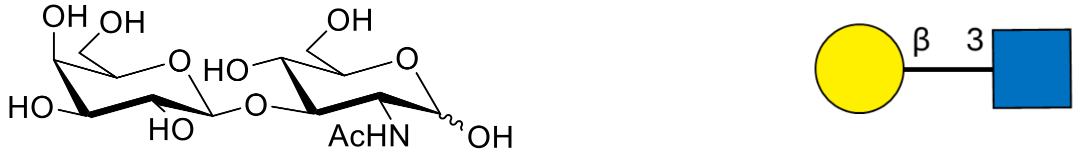

Figure S40. Gal-β-1,3-GlcNAc

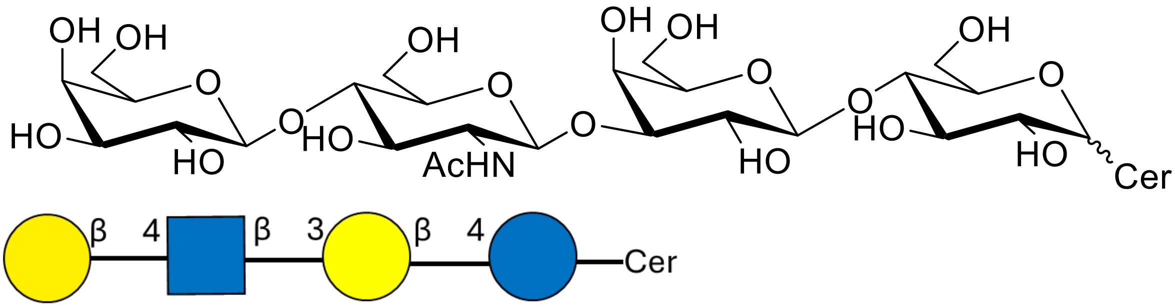

Figure S41. Neolactotetraosylceramide

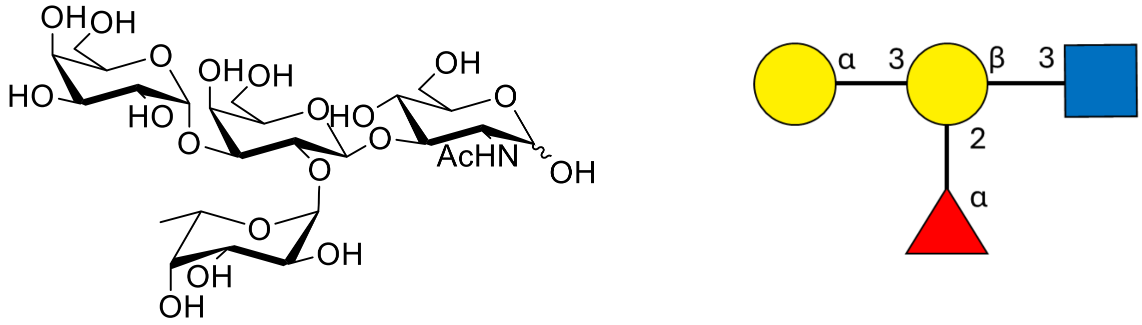

Figure S42. Gal-α-1,3-(Fuc-α-1,2)-Gal-β-1,3-GlcNAc

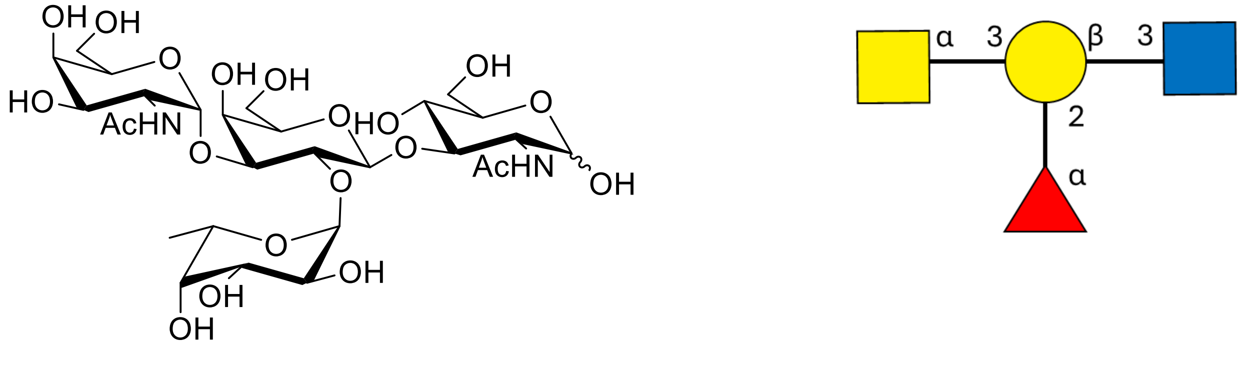

Figure S43. GalNAc-α-1,3-(Fuc-α-1,2)-Gal-β-1,3-GlcNAc

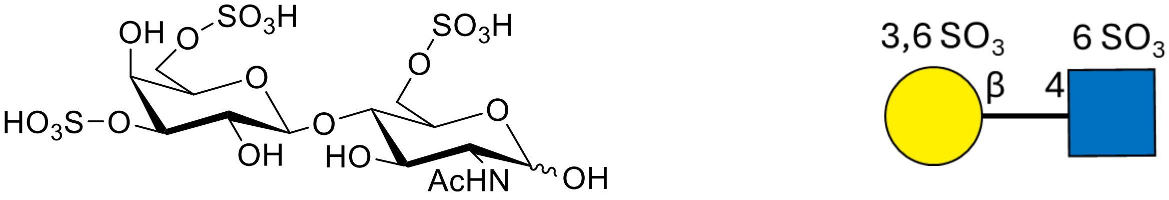

Figure S44. Gal-β-1,4-GlcNAc sulfated at positions 3', 6' and 6

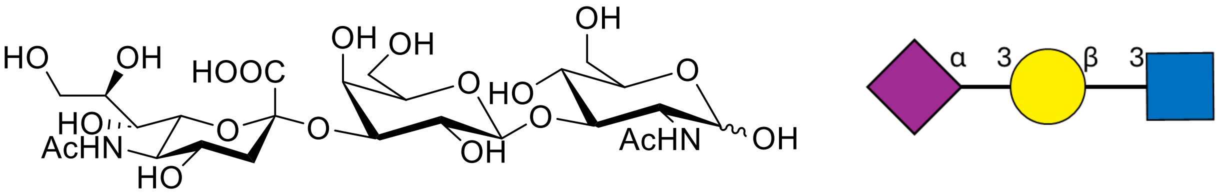

Figure S45. Neu5Ac-α-2,3-Gal-β-1,3-GlcNAc

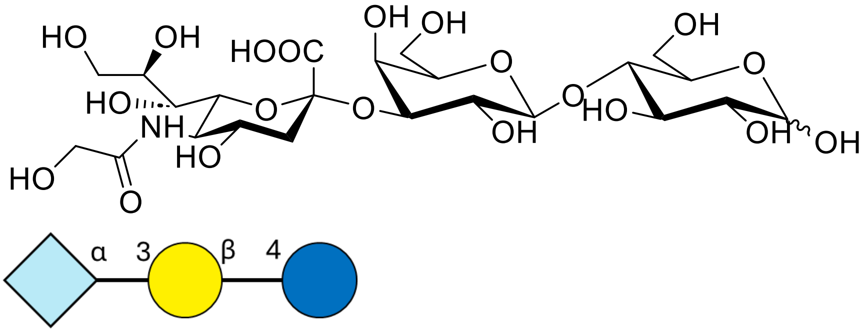

Figure S46. Neu5Gc-GM3

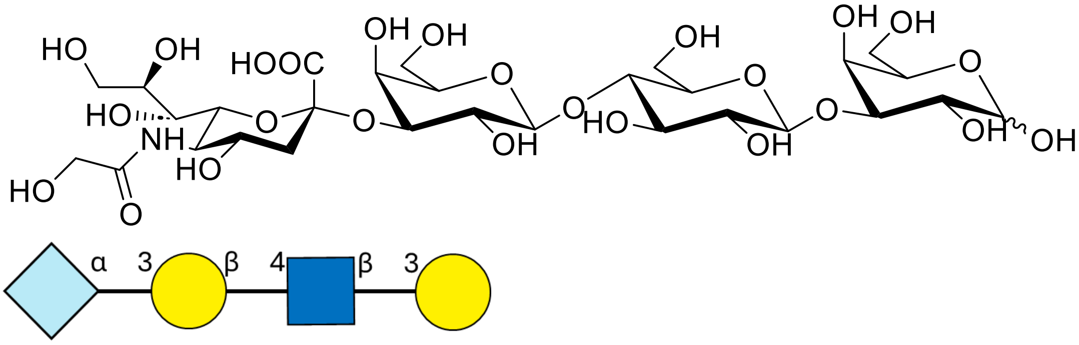

Figure S47. Neu5Gc-paragloboside

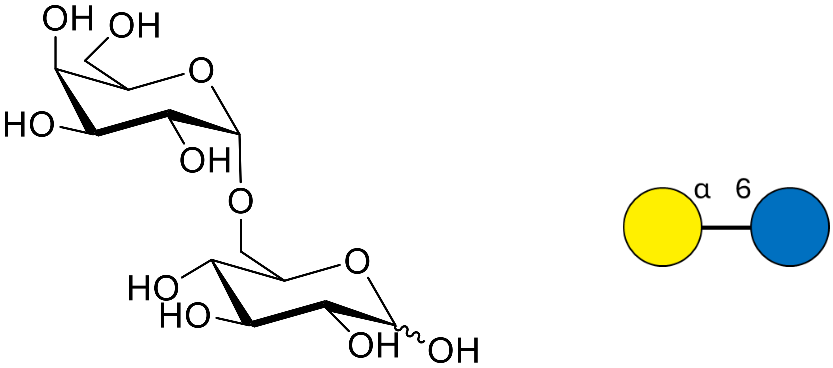

Figure S48. Gal-α-1,6-Glc

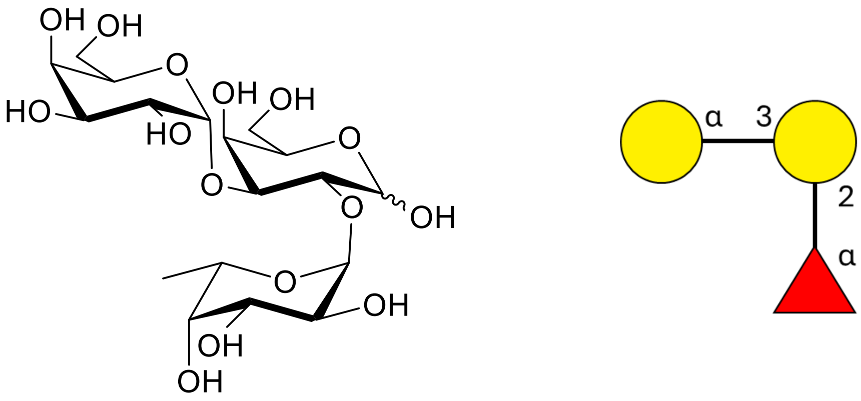

Figure S49. Blood group B trisaccharide

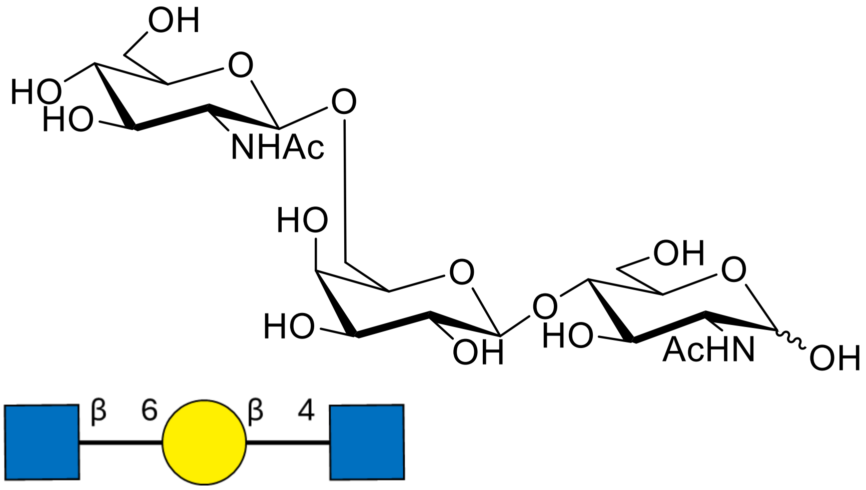

Figure S50. GlcNAc-β-1,6-Gal-β-1,4-GlcNAc

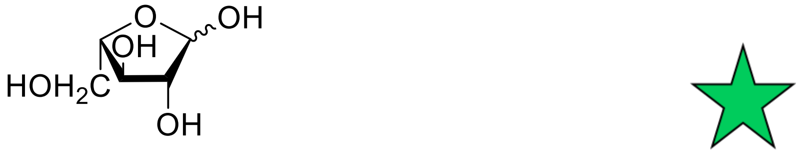

Figure S51. L-Arabinose

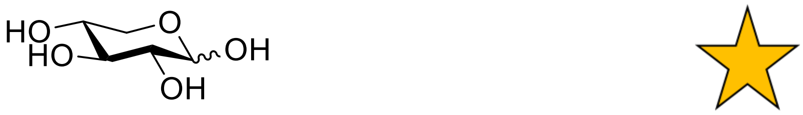

Figure S52. Xylose

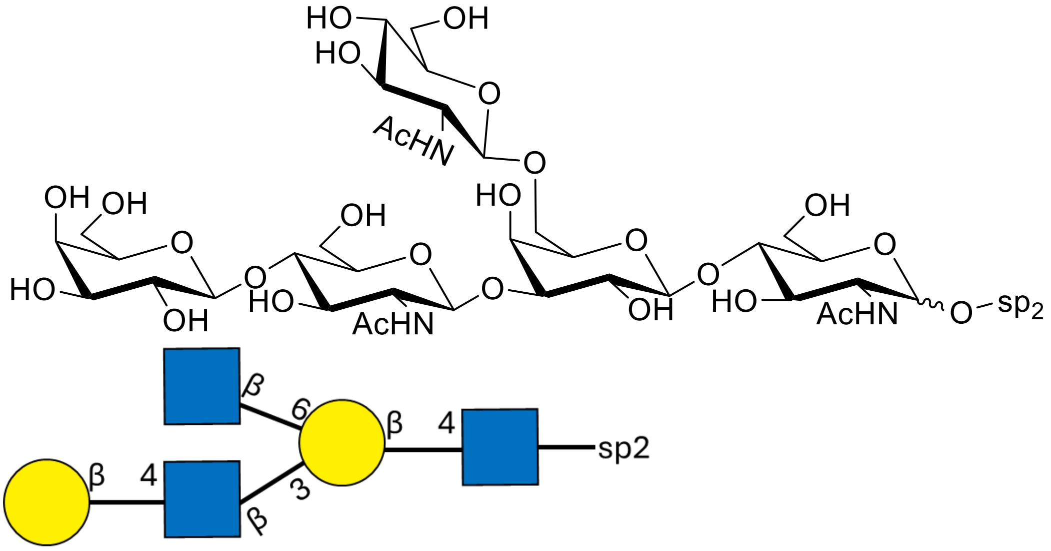

Figure S53. Gal-β-1,4-GlcNAc-β-1,3-(GlcNAc-β-1,6)-Gal-β-1,4-GlcNAc-sp2

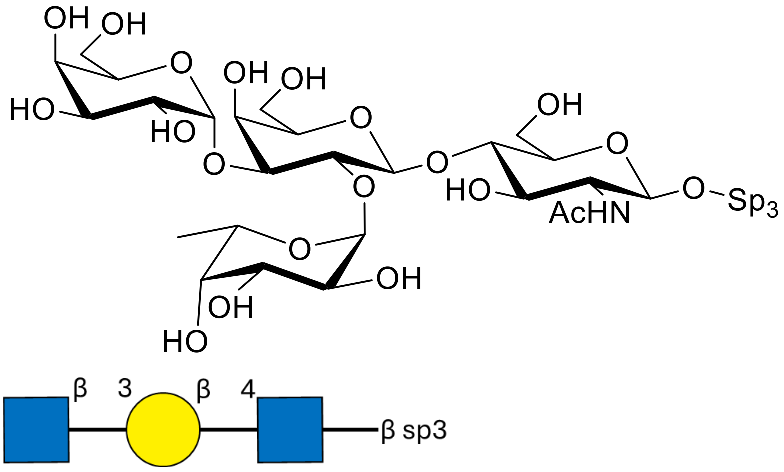

Figure S54. GlcNAc-β-1,3-Gal-β-1,4-GlcNAc-β-sp3

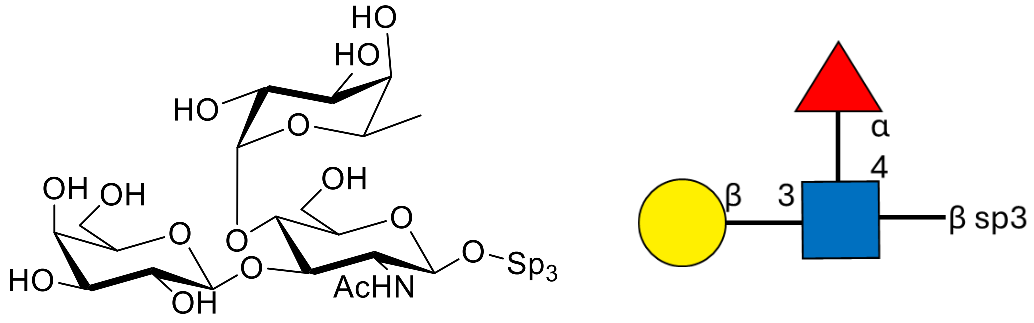

Figure S55. Gal-β-1,3-(Fuc-α-1,4)-GlcNAc-β-sp3

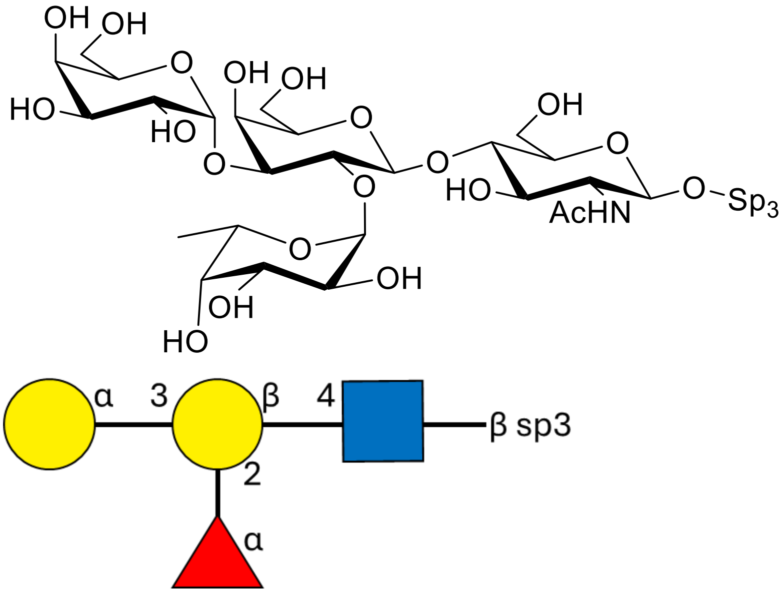

Figure S56. Fuc-α-1,2-(Gal-α-1,3)-Gal-β1,4-GlcNAc-β-sp3

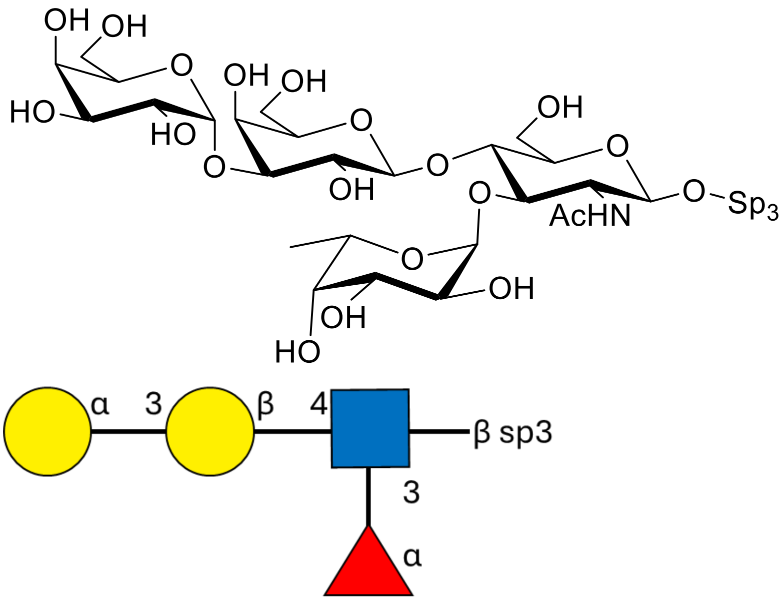

Figure S57. Fuc-α-1,3-(Gal-α-1,3-Gal-β1,4)-GlcNAc-β-sp3

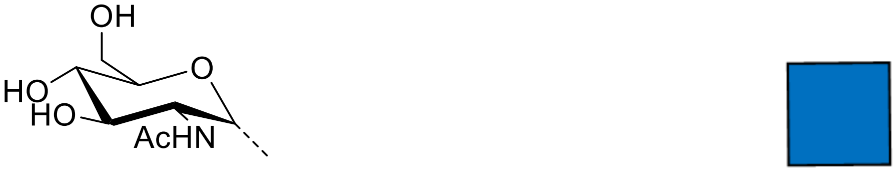

Figure S58. Terminal *N*-acetylglucosamine

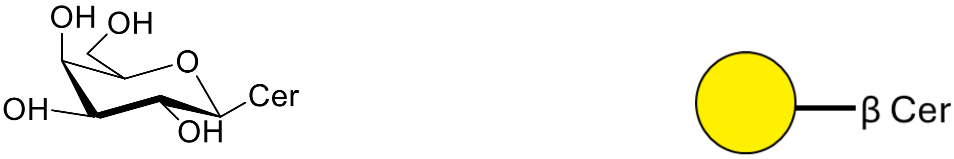

Figure S59. Galβ-1-1-Cer

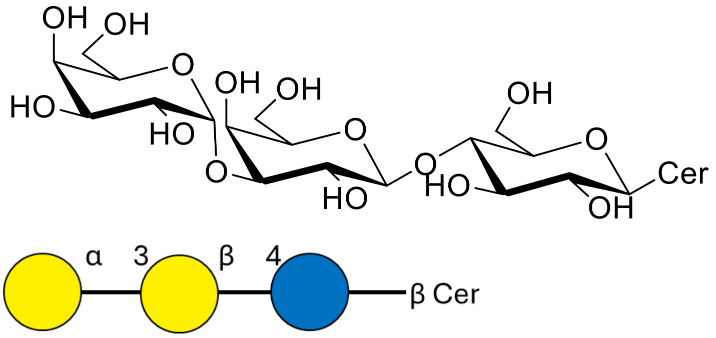

Figure S60. Isoglobotriaosylceramide

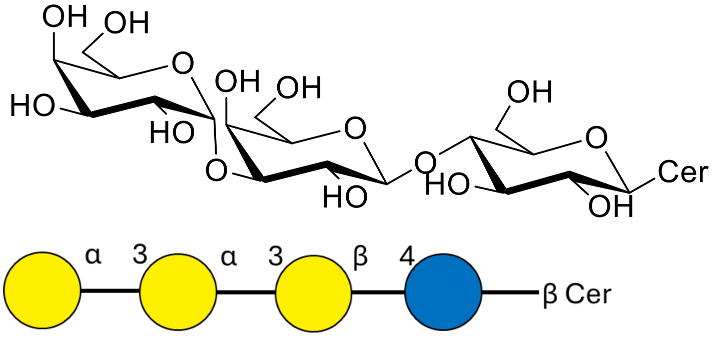

Figure S61. Gal-α-1,3-Gal-α-1,3-Gal-β-1,4-Glc-β-1-Cer

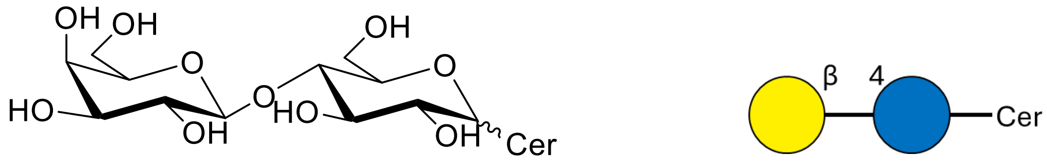

Figure S62. Lactosylceramide

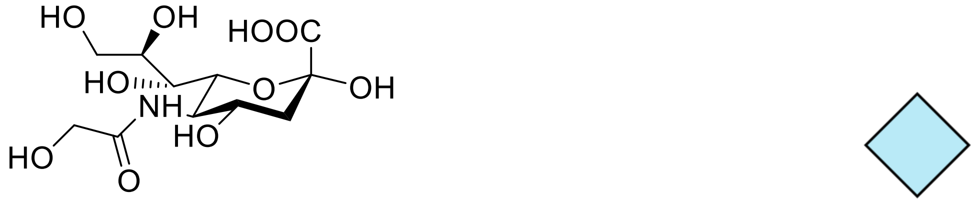

Figure S63. *N*-Glycolylneuraminic acid

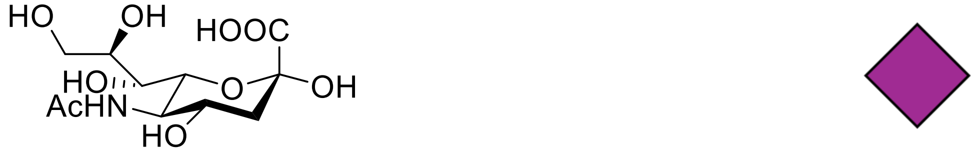

Figure S64. *N*-Acetylneuraminic acid

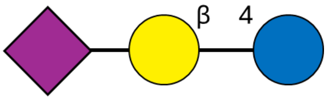

Figure S65. Neu5Ac-Lac

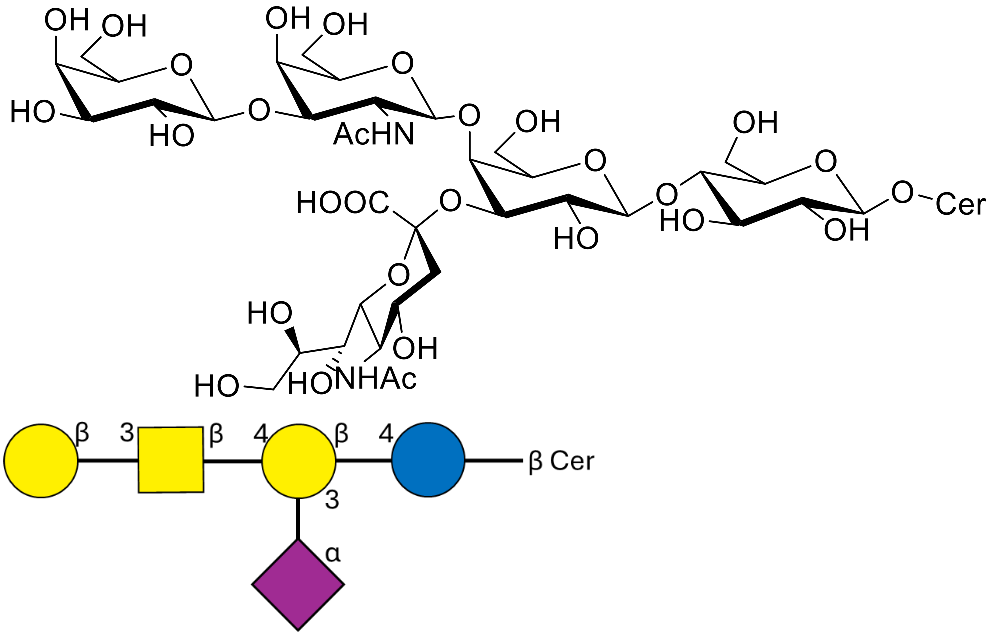

Figure S66. GM1

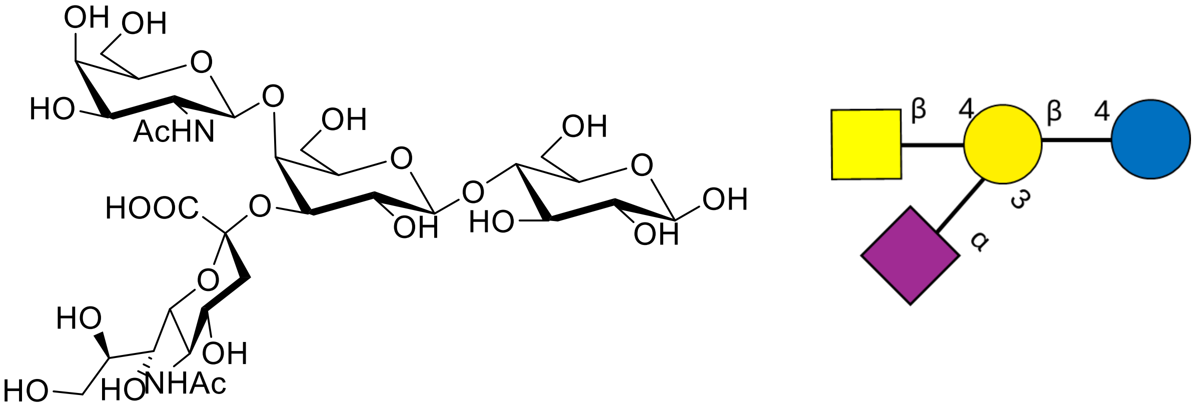

Figure S67. GM2

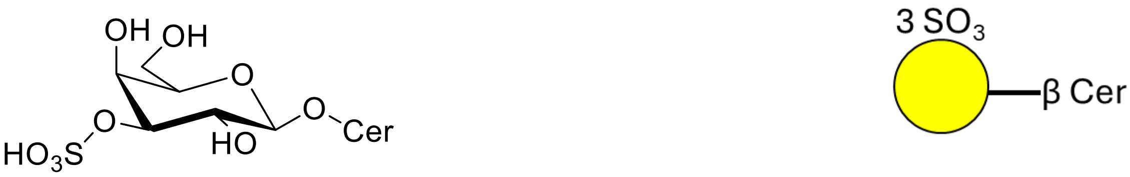

Figure S68. SO3-3-Gal-β-1-Cer

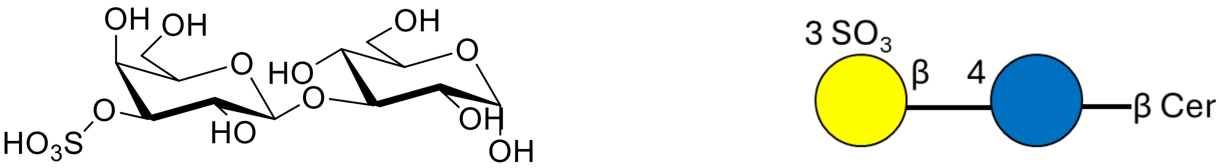

Figure S69. SO3-3-Gal-β-1,4-Glc-β-1-Cer

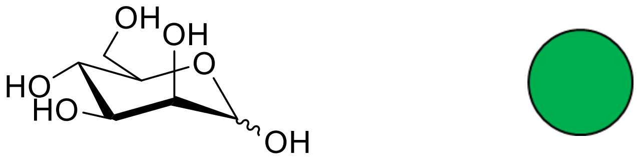

Figure S70. Mannose

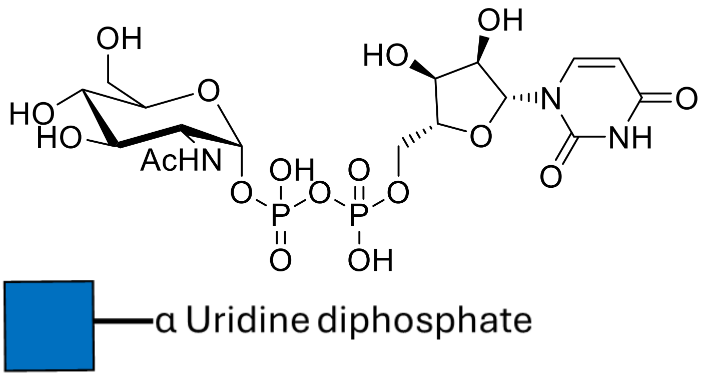

Figure S71. Uridine diphosphate *N*-acetylglucosamine

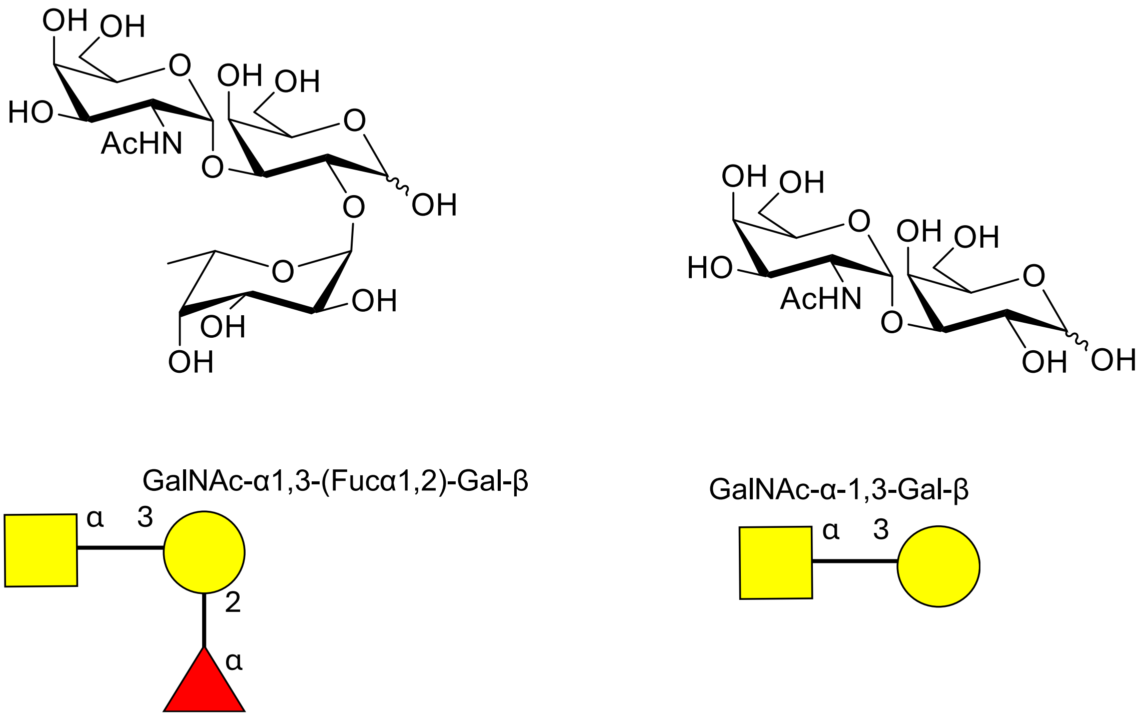

Figure S72. Blood Group A Antigens that bind EtpA

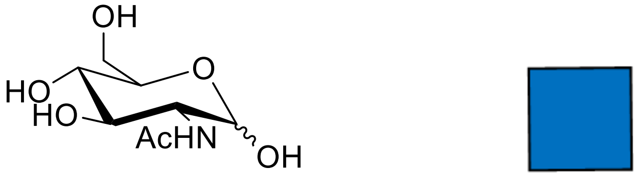

Figure S73. *N*-Acetylglucosamine

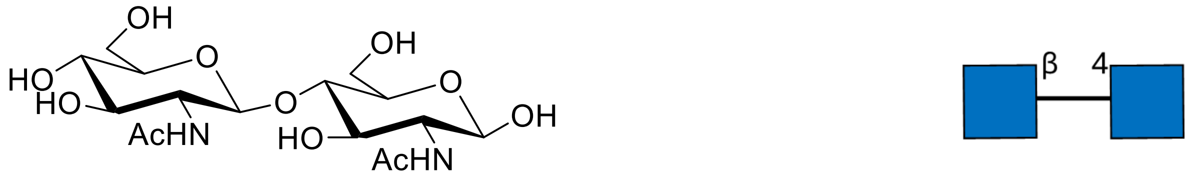

Figure S74. *N*,*N*'-Diacetylchitobiose

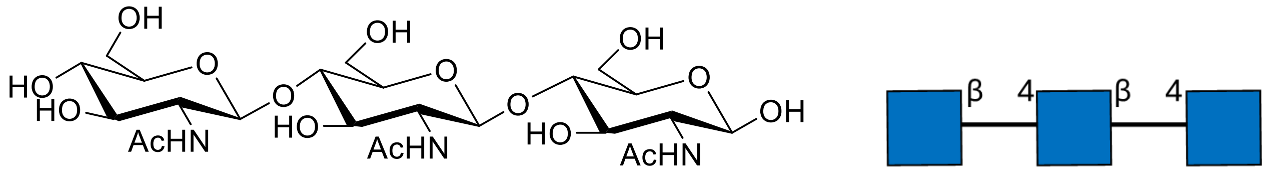

Figure S75. *N*,*N*',*N*''-Triacetylchitotriose
